# Reconstructing a spatially heterogeneous epidemic: Characterising the geographic spread of 2009 A/H1N1pdm infection in England

**DOI:** 10.1038/srep29004

**Published:** 2016-07-11

**Authors:** Paul J. Birrell, Xu-Sheng Zhang, Richard G. Pebody, Nigel J. Gay, Daniela De Angelis

**Affiliations:** 1 Medical Research Council Biostatistics Unit, Cambridge Insitute of Public Health, Forvie Site, Robinson Way, Cambridge Biomedical Campus, Cambridge CB2 0SR, UK; 2 Centre for Infectious Disease Surveillance and Control, Public Health England, 61 Colindale Avenue, London, NW9 5EQ, UK; 3 Fu Consulting, Hungerford, UK

## Abstract

Understanding how the geographic distribution of and movements within a population influence the spatial spread of infections is crucial for the design of interventions to curb transmission. Existing knowledge is typically based on results from simulation studies whereas analyses of real data remain sparse. The main difficulty in quantifying the spatial pattern of disease spread is the paucity of available data together with the challenge of incorporating optimally the limited information into models of disease transmission. To address this challenge the role of routine migration on the spatial pattern of infection during the epidemic of 2009 pandemic influenza in England is investigated here through two modelling approaches: parallel-region models, where epidemics in different regions are assumed to occur in isolation with shared characteristics; and meta-region models where inter-region transmission is expressed as a function of the commuter flux between regions. Results highlight that the significantly less computationally demanding parallel-region approach is sufficiently flexible to capture the underlying dynamics. This suggests that inter-region movement is either inaccurately characterized by the available commuting data or insignificant once its initial impact on transmission has subsided.

Transmission and spread of infectious diseases depend, in part, on the frequency with which infected people come into contact with susceptible individuals. Understanding the spatial heterogeneity of transmission and spread from one location to another is crucial for policymakers to allocate healthcare resources and to design effective control strategies. This has been illustrated for influenza by simulation studies using spatial models of transmission, at global^1^,^2^,^3^, continental^4^ and national levels^5^,^6^,^7^,^8^, providing useful information on the role of spatial factors and control measures on the spread of infection. Estimation of such roles from data, as opposed to exploring them through simulation, is much more complex and is typically constrained by a paucity of data to identify the spatial dynamics of infection. Recently, finely resolved spatial and temporal influenza data has been used to estimate the spread of infection during the autumn 2009 wave of A/H1N1pdm influenza in the US, finding that it was dominated by short-range transmission events^9^. This type of study is, however, rare and hard evidence of how heterogeneity in demographic processes can influence transmission remains limited^10^,^11^.

The global 2009 A/H1N1pdm outbreak gave rise to an epidemic in England characterised by two distinct waves of infection, occurring, atypically, in summer and in late autumn of 2009, outside of the traditional flu season. During this outbreak, sero-epidemiological data showed significant heterogeneity in the timing of the pandemic across the various government office regions (GORs)^12^,^13^. This information, alongside a number of complementary data streams, was used to disentangle the complicated processes of transmission dynamics and disease reporting for London^14^. London was treated as a closed system fed by an initial number of infectious individuals, leading to two distinct epidemic waves with the peak times of infection mainly driven by the influence of school holidays on contact patterns. Using related data, a SEIR epidemic system was developed to estimate transmission in the whole of England^15^. The sampling of both the serological and, in particular, the virological data used was very uneven across England, being concentrated in regions of particularly high disease transmission. To provide a meaningful local description of the epidemic using data of this type, it is important to aggregate data at a spatial resolution that gives sufficiently large within-region sample sizes while still making assumptions of homogeneous mixing within spatial units justifiable.

Here we extend previous work^14^ by developing multi-region modelling approaches to investigate spatial transmission and the possible role of inter-region movements in the spread of infection in England. We consider two types of model: a parallel-region (PR) model, where epidemics in different regions are assumed to occur in isolation, but are described by models with some common parameters; and a meta-region (MR) model, where the epidemic acts on a single population, stratified by age and region, with the populations from each stratum interacting through commuter flux. We use these approaches to explore the spread of the first two waves of 2009 pandemic influenza across England, estimating their dynamic characteristics based on a range of epidemic surveillance data including general practitioner (GP) consultations, seropositivity, virological positivity and case confirmations (see Fig. 1 for the London data).

## Results

We have divided England into four regions: London, West Midlands, the North and the South (see Materials and Methods: Data). These four regions are assumed either to be non-interacting, spatially disjoint populations (PR model) or to interact with each other via the movements of commuters within a single population subdivided into strata defined by the regions (MR model). Within each population, the model is as described in detail in Birrell *et al*.^14^. Briefly, the model includes a transmission component that feeds newly infected individuals into a disease and reporting component describing the progress of infected individuals to symptomatic illness and the mechanisms through which this illness is reported to the healthcare system. Table 1 itemises the model parameters to be estimated, specifying their spatial heterogeneity under both approaches. In expanding the model of Birrell *et al*.^14^ to the MR model, there are a number of modelling choices to be made: the handling of density dependent effects on transmission; the distribution of the seeding of infectious individuals; and the assumption of fixed versus random commuting. These issues are discussed in depth in the Methods: Modelling Approaches section and references therein. The MR model results presented here assume a model variant that has density dependence according to the size of the regional population; an assumption of random commuting (where every member of the population is assumed equally likely to commute on any given day); and an empirical-based seeding for the number of infections prior to the start date of May 1st 2009, the so-called ‘extended empirical’ seeding described in Supplementary Information (SI) Section 1.4.3.

### Reconstructing the epidemic

The two models are sufficiently flexible to reproduce the two epidemic waves of 2009 pandemic influenza (Fig. 2, SI Figs S2–S5). The estimated epidemic in the North is consistent across models. London and the West Midlands are characterized by bigger first waves of infection (and subsequently smaller second waves) under the PR model, with the opposite being estimated for the South. This is apparent from the height of the peaks in Fig. 2 and the attack rates in Table 2. Peak timings in both waves of infection are the same under both modelling approaches and coincide with the start of a school holiday. The exception to this is the second wave in the West Midlands, the region with the lowest estimated attack rate. Here, a sufficient supply of susceptible individuals remains in the population to allow transmission to increase once more (albeit briefly) when the schools re-opened after the short holiday. For comparison with other studies, SI Table S4 breaks these down into age-stratified results summarised at both regional and national levels.

### Estimated epidemic characteristics

Table 3 presents estimates of some key transmission parameters under each model. Estimates for the reproductive number (*R*_0_) are centred on 1.8, consistent across modelling approaches and, in the PR model, across regions. Similarly, the estimates for the other transmission parameters are robust to the model specification (note the overlapping nature of the credible intervals (CrIs) in Table 3). In particular, estimates for *m*_1_ indicate that the POLYMOD-estimated contact rates involving at least one adult had to be down-weighted by a factor of between 0.57 and 0.62. Estimates for *m*_3_ indicate instead that the summer school holiday period led to a rather dramatic decline in effective contact rates among 5–14 year-olds, with the resulting rate being less than 1% of that during school terms. By comparing *m*_5_ with *m*_3_, it is seen that both models identify a much weaker effect for the other, shorter, school holidays, their shorter duration causing milder disruption to usual contact patterns.

### Model performance

The overall fit of the PR model is superior to that of the MR models considered. The PR model has a greater flexibility due to the greater number of free model parameters to be estimated: *R*_0_ and the initial level of infectiousness, *I*_0_, are each described by four region-specific parameters, quantities represented by just one global parameter in the MR model. However, even taking this into account, there is enough evidence (see the bottom two rows of SI Table S3) in favour of the PR model to suggest a significant improvement in the fit of the model. This compounds the practical benefit of the PR model being faster to implement; it is much more suited to parallel computation and only requires the calculation of the spectral radius of (7 × 7) next generation matrices as opposed to (28 × 28) matrices for the MR model.

### Sensitivity to specification of the meta-region model

So far results from a ‘best’ variant of the MR model have been presented. We have also investigated a number of alternative parameterisations for this approach and the set of models considered is discussed further in Methods: Modelling Approaches. Density dependence, not a major consideration in the PR model, is best accounted for in the MR model by replacing *N*_ra_ with *N*_r_ = Σ_a_*N*_ra_. in Equation (3) and setting *α* = 1. This represents density dependent effects that are determined by the size of the regional population and not the population sizes of the individual stratum. Additionally, the model performs better when given the ‘extended empirical’ seeding (see SI Section 1.4.3) as opposed to any of those based purely on disease-free equilibria as done elsewhere^14^. The differences observed in the fit of the model when comparing the assumption of a fixed group of commuters versus random commuting are highly sensitive to the precise parameterisation. In the preferred model presented here, there is little difference between the two hypotheses, suggesting that the crude random commuting assumption is adequate enough. With no consistent difference in the model fit, random commuting requires fewer evaluations of SI Equations S1 and S4 and is, therefore, computationally more efficient to implement.

## Discussion

We have conducted a coherent, unified, Bayesian statistical analysis of multiple streams of epidemic surveillance data from the 2009 A/H1N1pdm outbreak in England, producing age and region stratified epidemic reconstructions (with associated uncertainty) and robust estimates for some key parameters of the transmission process. In particular, we have assessed the strengths and weaknesses of two different approaches in the presence of strong regional heterogeneity in the spread of influenza infection. Both the PR and MR models fit adequately well to the various data sources, with highly comparable estimates for both model parameters and epidemic characteristics that are consistent with existing literature. Results highlight that the PR approach is parsimonious yet sufficiently flexible to capture the underlying dynamics. This may imply that the impacts of inter-regional movement are either inaccurately characterized by the available commuting data or not significant beyond a transient initial forcing.

Spatial heterogeneity in transmission arising from the interaction between regional populations, is incorporated in the MR model through commuting flows. Therefore, the MR model has the capacity to predict the spatial spread of influenza infection early in an epidemic, as infection is transmitted according to these flows. The PR model is ‘non-parametric’, in the sense that the parameters representing the epidemic growth and initial seeding of infectiousness in each region are estimated without being subject to any parametric assumption. The timing of the epidemic waves in each region is highly dependent on these parameters and estimation of the respective epidemic curves requires some epidemic activity in all regions. Early in a pandemic, therefore, the MR approach is more useful in a predictive modelling setting. However, as discussed in the Results section, the MR approach involves an additional computational burden that limits its use as a tool for timely epidemic tracking as data accumulate over time.

The non-parametric nature of the spatial variation in transmission of the PR model confers on it greater flexibility, lending it an advantage when it comes to epidemic reconstruction, observed here in a significant improvement in model fit. An additional advantage is that this modelling approach does not rely on the validity of the commuter data to describe the spread of infection, nor does it rely on the assumptions that individuals maintain routine commuting behaviour regardless of infection status.

Despite the spatial variation in epidemic growth rates, the PR model provides estimates for *R*_0_ that are consistent across regions (see Table 3, caption). Therefore, the spatial heterogeneity in infection is being accounted for through the initial seeding of infectiousness. It has been seen elsewhere that long-range interactions have a declining role in the spread of a pandemic once infection is widespread in each region^3^,^8^,^10^. This is exacerbated for A/H1N1pdm influenza as school-age children, the demographic group most affected, do not contribute to commuter flows. Therefore, an improved fit of the MR model would most effectively be achieved through more flexible estimation of the initial seeding of infectiousness.

One variant of the MR model investigated here involved the stratification of the population within each region into commuters and non-commuters^16^. This has the effect of assuming each region contains a fixed sub-population of individuals who commute daily. This yields no consistent improvement in model performance, whilst increasing even further the computational cost. Factoring in the ‘random’ movements of casual and occasional travellers, which has been quoted to potentially increase the rate of transmission between regions by 25%^8^, would involve further computational burden and is particularly difficult to implement in an inferential setting without appropriate auxiliary information (*e.g.* if the census data contained information on the purpose of travel). The MR model could be made more realistic and detailed by assuming that a proportion of those with symptomatic illness may not travel^3^, or that asymptomatic illness is less infectious^17^. However, consideration of such factors would only lessen the contribution of long-range transmission, leaving conclusions unchanged.

There are a number of studies that provide estimates for incidence and attack rates in England during the two waves of 2009 A/H1N1pdm. However, estimates stratified by age and region are not publicly available. Our attack rates estimates, when aggregated to a national level, are highly consistent with those published elsewhere, based largely on the serological data used here^13^,^18^. A further study^19^ provides comparable overall incidence, but with a more even distribution of infection over the two waves and increased levels of infection in the older age groups. In this work the lower cumulative incidence in the first wave may be attributable to the parameter that measures the decrease in the rate of effective contact among 5–14 year-olds suggesting a drop of over 99%. Averaged across all age-groups, this represents a drop in *R*_0_ of between 43% (in London) and 50% (in the South). To compare, He *et al*.^20^ record a 28% fall in transmissibility during a school holiday period. These represent drops from a baseline *R*_0_, estimated to be in the region of 1.8, a value corroborated in literature^21^.

The modelling approaches presented here have great potential for use in a future pandemic, and will form a key component of the pandemic response protocol of the responsible public health body in England, Public Health England. Here, GP consultations have been used to inform the model, but in practice any time series of count data related to infection incidence could be used to inform the pattern of infection over time. Such data could alternatively come from hospital admittances, absenteeism^22^, antiviral prescriptions etc. Serological data underpin the scale of infection and in their absence the full scale of the epidemic cannot be accurately estimated until the epidemic has been fully observed^14^. If the count data are not pathogen-specific and hence contaminated, then some virological data are required to identify the signal due to the pandemic. All pandemic data sources discussed here do not need to cover the whole population. Data can be included provided that there is information on the covered fraction of the population and that any bias in this coverage is well understood.

Since 2009 in the UK there has been an investment in improving the quality of the surveillance data available in the event of a pandemic. Such improvements can only enhance the utility of the evidence synthesis model presented here. The prompt availability of hospitalisation and intensive care unit admission data could remove (at least in the early stages of a pandemic) the dependence on noisy GP data that are influenced by fluctuating healthcare-seeking behaviours of the public. These noisy GP data require attendant virological swabbing data, the positivity of which wanes over time from symptom onset. This sensitivity is crudely accounted for here by omitting any swabs taken more than five days since onset. Methods for incorporating the uncertainty in the swab results into this modelling framework would be valuable. The serological data come from the analysis of blood sera samples taken from patients admitted to hospital for a variety of non-respiratory reasons. It is unclear if this convenience sampling approach could lead to bias. Furthermore, the relationship between the recorded titre values and the presence of an immunological response is imprecise and uncertain^13^. Joint modelling of serological microarray data with syndromic surveillance data to reconstruct an epidemic has incorporated this imprecision^23^, but in this exercise the data do not have sufficient resolution to clearly partition long-standing immunity from recent infection and from susceptibility.

To summarise, using a Bayesian statistical framework, the PR model is found to be sufficiently flexible to provide a good fit to data and is quick to implement as it includes lower dimension contact matrices and, particularly, as model code can be easily parallelised. Reassuringly, it also provided concurring estimates for the basic reproductive number (*R*_0_) across the regions, in agreement with the MR approach. However, the PR model can provide little insight on inter-region transmission and the determinants of spatial heterogeneity in the spread of infection because of its simple structure. In a situation where school-age children are the main agents of transmission and baseline transmissibility is not high, spatial models that concentrate on local transmission, like the PR model, provide a powerful and timely tool for use by public health services, helping to inform effective control and containment measures.

## Methods

### Data

The epidemic dynamics are reconstructed on the basis of a suite of epidemic surveillance data available during the 2009 pandemic. This includes counts of non-disease specific illness in the form of GP consultations for influenza-like illness (ILI) and seroepidemiological and virological swabbing data. A full description of these data sources has been published in the SI of Birrell *et al*.^14^, so we only summarise them briefly here.

**Figure 1:**
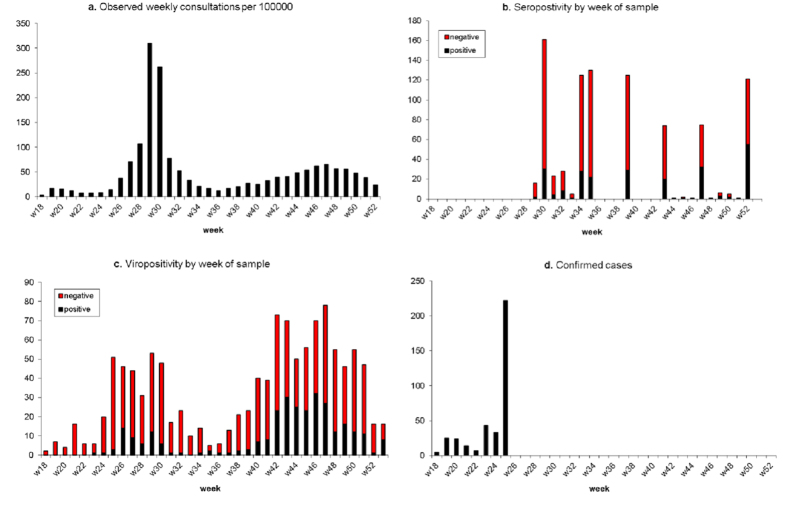
The four different data types used in the modelling presented for London: (**a**) Weekly GP consultation counts; (**b**) Weekly counts of blood sera samples tested, and the proportion that test positive; (**c**) Weekly counts of swab samples collected for virological testing and the proportion positive; (**d**) Numbers of A/H1N1pdm cases confirmed in the early part of the epidemic, by week.

**Figure 2:**
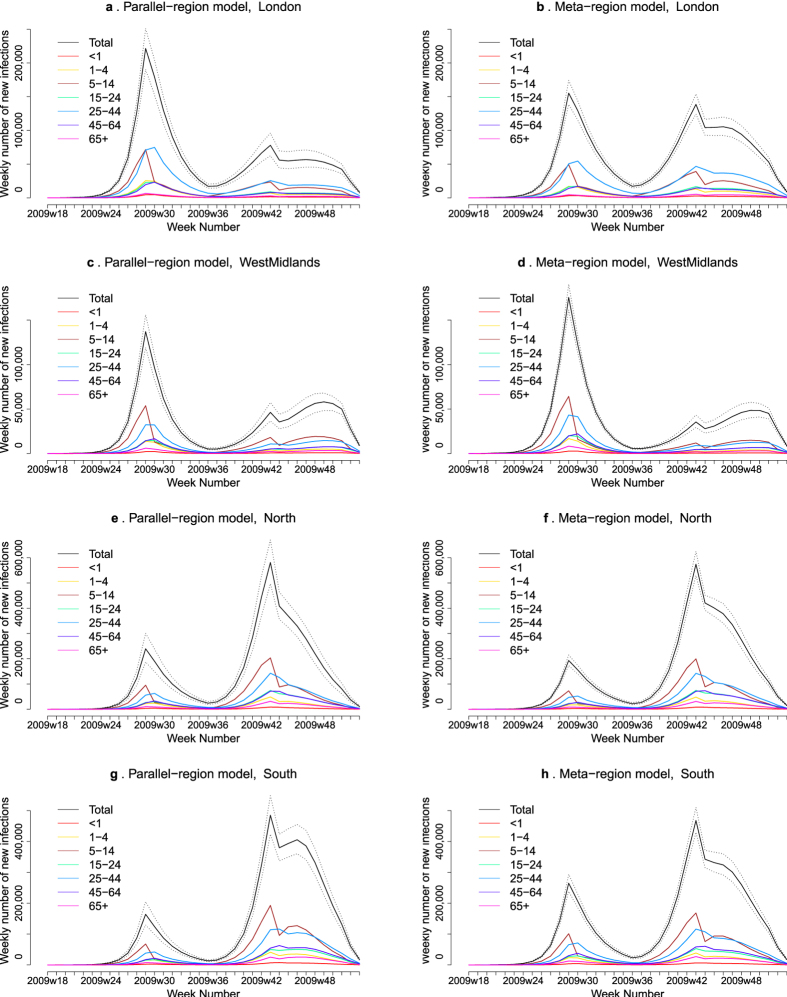
Estimated weekly number of new A/H1N1pdm infections by region (row) under the PR model (left column) and the MR model (right column). The solid black lines represent incidence summed over age groups with an associated 95% Credible Interval (CrI) (dashed lines). Different colours represent posterior medians for the age group-specific infection incidence.

The ILI consultation data come from sentinel GP surveillance, providing daily counts of consultations in participating practices, stratified by age and region, as well as daily denominators giving the fraction of the population (typically >50%) covered by the scheme^24^. Virological swabbing provide a (short) time series of case confirmation data derived as a result of contact tracing carried out on some of the early identified cases^25^ and a longer companion dataset to the GP surveillance data on the proportion of GP ILI consultations testing swab-positive for the pandemic pathogen^26^,^27^. Additionally, infrequent batches of serological data provide information on the proportion of the wider population carrying protective antibodies, assumed to be indicative of the level of cumulative infection up until two weeks (the length of time allowed for antibodies to establish within host) prior to the time of sample^12^. With the exception of the case confirmation data, all datasets run from 1st May to 31st December, 2009, giving 245 days of consecutive data (see Fig. 1 for a presentation of this data from London).

To ensure large enough sample sizes, we divide England into four regions: two smaller regions that exhibited a significant first wave of infection, London and the West Midlands; and two regions that cover a larger area, labelled North (combining the North-West, North-East, Yorkshire and Humberside, and the East Midlands GORs^28^) and South (combing the East of England, South-East and South-West GORs). Commuting data have been extracted from the UK 2001 census^29^. The census provides an estimated number of people of all ages >15 years moving between each of our regions on the date of the survey (including those that do not move). The age-specific commuter matrices are shown in SI Table S1. The population size and structure over seven age groups (<1, 1–4, 5–14, 15–24, 25–44, 45–64, >64 years) in each of the four regions have been extracted from mid-year population estimates released by the Office of National Statistics^30^.

### Modelling Approaches

To model the spatial spread of infection, the population is divided into strata defined by region and age pairs, (*r*, *a*), *r* = 1, …, *R*; *a* = 1, …, *A*. Each stratum is assigned an index *j* = *a* + *A*(*r* − 1), *j* = 1, …, *RA*. The infection status of the population within stratum *j* at discrete-times *t*_*n*_ = *nδt* is described by a deterministic SEEIIR system of difference equations:


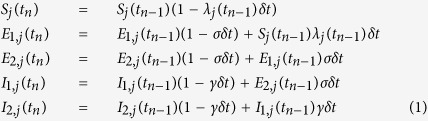


for *n* = 1 … *T* and suitably small *δt* (here taken to be 0.5 days). Parameters *σ* and *γ* are related to the mean duration of latent and infectious infection, *d*_*L*_ and *d*_*I*_ respectively via *σ* = 2/*d*_*L*_, *γ* = 2/*d*_*I*_ and the force of infection, *λ*_*j*_(*t*_*n*_), is expressed through the Reed-Frost formulation


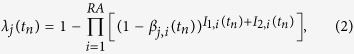


where the (*j*, *i*)^th^ entry of the time-varying (*RA* × *RA*) matrix ***β***(*t*_*n*_) gives the infection pressure exerted on a susceptible individual within stratum *j* by a single infectious individual in stratum *i*. The structure of the matrix ***β***(*t*_*n*_) depends on assumptions governing spatial heterogeneity in interpersonal contact rates and the transmissibility of infection across different strata. Two approaches are formulated to handle the spatial heterogeneity: parallel-region and meta-region modelling (see below and SI Sections 1.3-4 for greater detail). Both formulations can be parameterised in a similar fashion, with slight differences in the spatial variation of some parameters as illustrated in Table 1.

#### Parallel-Region (PR) Modelling

The PR model assumes that infectious individuals exert negligible infectious pressure on individuals in any of the other regions and the transmission dynamics in each region are considered independent of the dynamics occurring elsewhere. This results in parallel, single-region, epidemics linked through the borrowing of strength between some parameters (*e.g.* the background model, see Table 1) or the sharing of some common parameters (*e.g.* the proportion of symptomatic infections, Table 1). Within each of the four English regions the epidemic dynamics are governed by Equations (1) and (2) with *R* = 1 and *A* = 7. The strata are then simply defined by age groups and the, now regionally-dependent, (*A* × *A*) infection rate matrix is given by:


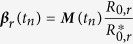


where ***M***(*t*_*n*_) = {*M*_*a*,*b*_(*t*_*n*_)} is a matrix of relative infective contact rates between individual of age groups *a* and *b* derived from POLYMOD data^31^ and the contact parameters, *m*_*k*_, *k* = 1, …, 5 (as described elsewhere^14^). This approach allows estimation of region-specific reproduction numbers, *R*_0,*r*_ (via the epidemic growth rates *ψ*_*r*_, see SI Section 1.3.1 and Equation S6). The 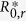 denote the dominant eigenvalues of the next generation matrices 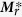 which has (*a*, *b*)^th^ entry given by *N*_*r*,*a*_ × *M*_*a*,*b*_(0) × *d*_*I*_, where *N*_*r*,*a*_ is the resident population size of people in age group *a* in region *r*.

Compared to the London study^14^, some minor amendments have been made to the model, including the addition of a day-of-the-week effect on the reporting of ILI (see SI Section 1.2.2) and the expansion of the background model, made feasible due to the integration of a spatial dimension in the modelling (see SI Section 1.2.1). Parameters that represent biological characteristics of the virus (mean infectious period, proportion symptomatic) are assumed to be consistent across all regions. Additionally, the contact parameters exhibit no regional variation. As well as the exponential growth rates, the initial levels of infectiousness (*I*_0,*r*_) are allowed to vary among regions, as they are a function of the regional population as well as of the virus, and can account for the different timing of the pandemic activity in each region. Spatial heterogeneities of model parameters are given in Table 1.

#### Meta-Region (MR) Modelling

In the meta-region modelling approach the four regions are connected by commuter flows into one system. The number of strata are defined by setting *R* = 4 and *A* = 7 and the resulting (28 × 28) contact matrix is denoted by Π. The (*j*, *i*)^th^ entry of Π, where *j* = *a* + *A*(*r* − 1), as above, and *i* = *b* + *A*(*s* − 1) represents the generic region/age strata (*s*, *b*), is as follows:


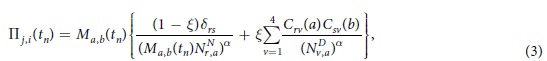


and the infection rate matrix has entries


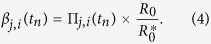


Matrices ***C***(*a*) in Equation (3) have entries *C*_*rs*_(*a*) representing the proportion of age group *a* resident in region *r* that commute into region *s* on any given day (see SI Table S1). The 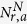 are the population sizes of stratum (*r*, *a*) at night (*i.e.* the size of the resident population) and 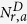 are the day population sizes (see SI Section 1.4.2), the adjusted population sizes after commuter movements have occurred; and *ξ* is the proportion of total time that a commuter actually spends in the commuting region. We set *ξ* = 5/14 on the basis of a daily average of five working days per week, being away from home for a half day when working. The exponent *α* takes values in [0, 1] with a value of 0 indicating frequency-driven transmission and 1, density-driven transmission. Finally, in Equation 4, 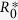 is again the dominant eigenvalue of a next generation matrix Π* which has entries 
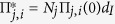
. As all strata interact and the meta-population cannot be broken down into isolated regions, there is only a single growth rate and hence a single value for the epidemic’s reproductive number, *R*_0_.

**Figure 3:**
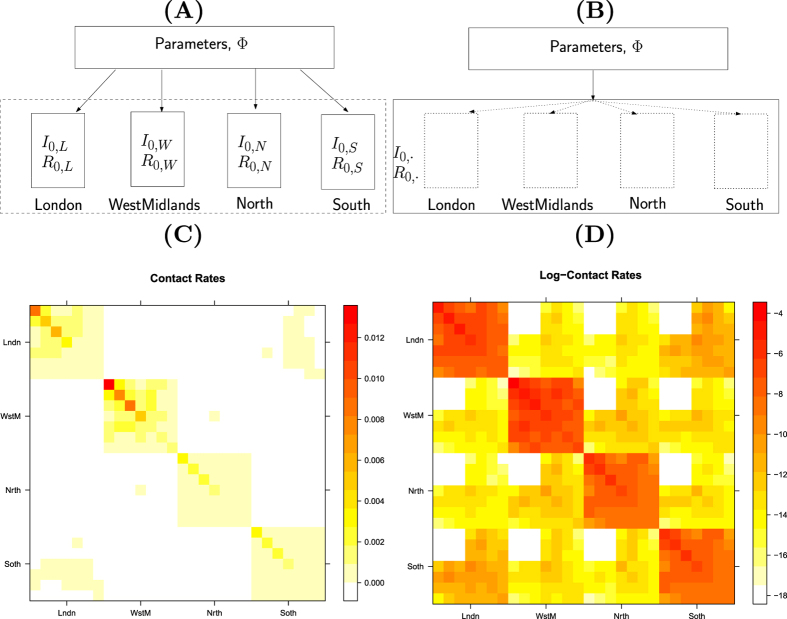
Model structures and contact patterns. Panels (**A,B**) are schematic diagrams illustrating the distinction between the PR and the MR models. (**C**,**D**) are heat maps for the contact matrices used in the MR model (with regional density dependence) based on the contact rates and log-contact rates respectively, showing their strong block diagonal structure. Red areas indicate higher rates of contact. The strata are organised within regions, so the block diagonal sections give rates of within-region contact. Diagonal elements give rates of within-strata (*i.e.* region and age) contact.

A structural comparison of the MR modelling to the PR modelling is illustrated in Fig. 3. There are a number of modelling considerations relevant to MR modelling that are not applicable to the PR approach, and these are discussed below.

##### Density Dependence

To test different variants of density dependence, the exponent *α* in Equation (3) is given three different values: 0 (the frequency dependent formulation), 0.5 and 1.0 (density dependent formulation). Also we consider replacing the population size (*N*_*r*,*a*_) of each stratum by the total population size (*N*_*r*_) of the containing region *r* to investigate the precise form of any density dependence (see SI Section 1.4.2).

##### Effect of seed construction

The near-block diagonal structure of the contact matrix (see Fig. 3(C,D)) results in convergence to a disease-free equilibrium being very slow (if it occurs at all). Any simulated epidemic from the meta-regional model is, therefore, qualitatively sensitive to the choice of the initial seeding of infection^7^. To identify an appropriate approach for generating such epidemic seeds, we consider three specifications labelled ‘nextgen’, ‘empirical’ and ‘extended empirical’, the details for which are given in SI Section 1.4.3.

##### Random commuting vs. fixed commuting

So far, in the MR model all members of a given stratum are assumed to be equally likely to commute, with the total proportion of commuters remaining the same. However, it may be more realistic to assume that the commuters are a fixed group of people. To account for this, adult groups in each region are further sub-divided into commuters and non-commuters, giving 11 strata per region, 44 in total. Although the total attack rate is insensitive to this further stratification, the peak times across regions are affected (see SI Section 1.4.4). Results suggest that the introduction of a fixed commuting population, however, improves model fit to the 2009 pandemic data across England only marginally (SI Table S3; cf.^16^).

### Parametric Inference

Assuming that the epidemic data are imperfectly observed, a Bayesian approach is used to estimate the unknown parameters. The posterior distributions of these parameters and various quantities of interest are derived through the combination of prior information and the likelihood function. The log-likelihood function includes information from four data components: the number of GP consultations, virological positivity, number of lab-confirmed cases and seropositivity. Full details of the likelihood function are given in SI Section 1.5.1.

#### Priors and Implementation

The Bayesian framework for statistical inference involves the specification of prior probability distributions for all model parameters. We have assumed a level of prior knowledge of the pandemic that was representative of the state of knowledge in 2009. Therefore, prior specifications are largely unchanged from those used in Birrell *et al*.^14^. In the PR model, where a single parameter is specified for each region, they are assumed *a priori* to be identically distributed according to the prior specified for London^14^. For the new parameters, such as day of week effect on reporting of GP consultations and the additional parameters of the background ILI consultation process, non-informative normal prior distributions are assumed (see SI Section 1.5.2 for technical details).

The Bayesian model is implemented using Markov Chain Monte Carlo^32^, using bespoke C++ code. The PR model, parallelised on an eight core machine takes c.15 hours to implement, as opposed to 60 hours for the MR model (with 28 strata). When assuming fixed commuting groups (*i.e.* 44 strata), this run-time doubles to approximately 120 hours. The code and input files used to generate the outputs in this paper, together with some dummy data can be found at www.mrc-bsu.cam.ac.uk/software/miscellaneous-software/. Requests for access to the data should be directed to: Richard.Pebody@phe.gov.uk.

## Additional Information

**How to cite this article**: Birrell, P. J. *et al*. Reconstructing a spatially heterogeneous epidemic: Characterising the geographic spread of 2009 A/H1N1pdm infection in England. *Sci. Rep.* **6**, 29004; doi: 10.1038/srep29004 (2016).

## Supplementary Material

Supplementary Information

## Figures and Tables

**Table 1:** Model parameters classified in the parallel-region (PR) and meta-region (MR) models as either being ‘spatial’, where region-specific, or ‘global’.

Parameter	Description	Model	
		PR	MR
***η***	Dispersion parameters for GP consultation	Spatial	Spatial
*d*_*I*_	Average duration of infectious period	Global	Global
*θ*	Proportion of infections that lead to ILI symptoms	Global	Global
*m*_*k*_, *k* = 1, …, 5	Parameters of the contact matrices*	Global	Global
Ψ_*r*_	Exponential growth rates	Spatial	Global
*ν*_*r*_	Initial number of infectives, log-transformed	Spatial	Global
***p***^(GP)^	Propensity of ILI patients to consult with their GP	Spatial	Spatial
***p***^(CC)^	Proportion of ILI patients who receive case confirmation	Spatial	Spatial
***β***_*B*_	Regression parameters determining rates of background ILI consultation	Spatial	Spatial
***κ***_*d*_	Day of the week effects on the reporting of consultations	Global	Global

^*^These parameters act as multipliers to elements of the POLYMOD contact matrices^31^: *m*_1_ is the factor by which contact rates involving adults are down-weighted; *m*_2_, *m*_3_ are reductions in contact rates among children aged 1–4 and 5–14 respectively in the over-summer school holiday; and *m*_4_, *m*_5_ are the corresponding reduction in contact rates for all other school holidays.

**Table 2:** Posterior median and 95% CrI for cumulative incidence of infection, number of cases (thousands) and attack rates, by region and by pandemic wave (May-August or September-December).

******	London	West Midlands	North	South
Parallel-region model				
*May-August*				
Infections	988 (958, 1,124)	525 (456, 600)	1,058 (839, 1,316)	692 (554, 854)
Cases	152 (123, 184)	80 (65, 98)	161 (121, 215)	105 (80, 139)
Attack rate (%)	13.2 (11.4, 14.9)	9.8 (8.5, 11.2)	5.6 (4.4, 6.9)	3.6 (2.9, 4.5)
*September-December*				
Infections	764 (641, 901)	571 (483, 656)	3,671 (3,379, 3,987)	3,750 (3,508, 4,021)
Cases	117 (91, 153)	87 (64, 115)	563 (462, 689)	576 (471, 697)
Attack rate (%)	10.1 (8.5, 11.9)	10.6 (9.0, 12.2)	19.3 (17.8, 21.0)	19.6 (18.3, 21.0)
Meta-region model				
*May-August*				
Infections	751 (674, 832)	669 (621, 718)	886 (792, 986)	1,150 (1,036, 1,270)
Cases	85 (74, 98)	76 (66, 88)	100 (87, 117)	130 (113, 151)
Attack rate (%)	9.9 (8.9, 11.0)	12.4 (11.5, 13.3)	4.7 (4.2, 5.2)	6.0 (5.4, 6.6)
*September-December*				
Infections	1,227 (1,227, 1,331)	477 (404, 559)	3,923 (3,721, 4,128)	3450 (3,255, 3,657)
Cases	139 (114, 172)	54 (42, 69)	446 (377, 532)	393 (328, 472)
Attack rate (%)	16.2 (14.9, 17.6)	8.9 (7.5, 10.4)	20.6 (19.6, 21.7)	18.0 (17.0, 19.0)

**Table 3:** Posterior median and 95% CrI for key parameters by model.

Parameter	Parallel-reg. model	Meta-reg. model
*R*_0_	–	1.81 (1.77, 1.84)
*d*_*I*_	3.47 (3.35, 3.59)	3.46 (3.34, 3.58)
*θ*	0.154 (0.126, 0.186)	0.114 (0.098, 0.134)
*m*_1_	0.569 (0.536, 0.605)	0.618 (0.584, 0.651)
*m*_2_	0.901 (0.610, 0.996)	0.666 (0.265, 0.740)
*m*_3_	0.007 (0.000, 0.032)	0.006 (0.000, 0.032)
*m*_4_	0.167 (0.008, 0.669)	0.214 (0.004, 0.909)
*m*_5_	0.446 (0.341, 0.557)	0.411 (0.291, 0.528)

Estimates of the reproduction number (*R*_0_) from the PR model are 1.79 (1.74, 1.83), 1.80 (1.76, 1.85), 1.82 (1.78, 1.87), 1.77 (1.73, 1.80) for London, West Midlands, North and South, respectively.
