# Supplementary material for: Reconstructing a spatially heterogeneous epidemic: Characterising the geographic spread of 2009 A/H1N1pdm infection in England
Source: Sci Rep. 2016 Jul 11;6:29004. doi: 10.1038/srep29004 (PMC4941410; doi:10.1038/srep29004)
Supplement: Supplementary Information [file srep29004-s1.pdf]

Reconstructing a spatially heterogeneous epidemic: Characterising the geographic spread of 2009 A/H1N1pdm infection in England

Paul J. Birrell[1,*,+], Xu-Sheng Zhang[2,+], Richard G. Pebody[2], Nigel J. Gay[3], and Daniela De Angelis[1,2]

[1]Medical Research Council Biostatistics Unit, Forvie Site, Robinson Way, Cambridge Biomedical Campus, Cambridge CB2 0SR, UK

[2]Centre for Infectious Disease Surveillance and Control, Public Health England, 61 Colindale Avenue, London, NW9 5EQ, UK

[3]Fu Consulting, Hungerford, UK

*Corresponding author: paul.birrell@mrc-bsu.cam.ac.uk

+these authors contributed equally to this work

Supplementary Information

Contents

S1. Materials and Methods 2

S1.1 The model of Birrell *et al.*[1] 2

S1.2 Model extensions 5

S1.2.1 Background consultations 5

S1.2.2 Day of the week reporting effects: 6

S1.2.3 Multi-region modelling 7

S1.3 Parallel-region model 8

S1.3.1 Parameterisation: 8

S1.3.2 Mixing 11

S1.3.3 Seeding 11

S1.4 Meta-region model 11

S1.4.1 Parameterisation 11

S1.4.2 Density dependence and mixing among regions 12

S1.4.3 Seeding 14

S1.4.4 Random Commuters vs. Fixed Commuters 15

S1.5 Inference 16
S1.5.1 Likelihood 17
S1.5.2 Priors 18
S2. Further Results 19
S2.1 The Estimated Epidemic and Goodness-of-fit 19
S3. The relationship between the exponential growth rate (ψ_r) and the reproductive number (R_0) in a heterogeneously-mixing population 21
References 28
Supplementary Tables 30
Supplementary Figures 34

S1. Materials and Methods

The basic, underlying model structure, like the data, is largely as described in SI Materials and Methods of Birrell *et al.*[1]. Here, we briefly outline the model dynamics in order to introduce a slight change in notation, and then we proceed to detail some model developments and detail two possible approaches to accommodating spatial heterogeneity in transmission.

S1.1 The model of Birrell *et al.*[1]

A deterministic SEIR transmission model (to be more exact, an SEEIIR model, so that the exposure (E) and infectious (I) states have Erlang distributed waiting times), outputs a time series of numbers of newly infected individuals, which, in turn, act as input into a disease reporting model. This model component describes the proportion of the new infections that will appear in each of the various surveillance data streams, and the time taken from the initial infection to the time of report of the symptomatic case.

In general, assume that a population has been divided up into J strata, and that we wish to monitor an epidemic over K time intervals $[t_{n-1}, t_n) = [(n-1)\delta t, n\delta t), n = 1, \dots, T$. Denote the state vector to be $\boldsymbol{v}(t) = \left(\boldsymbol{S}(t), \boldsymbol{E_1}(t), \boldsymbol{E_2}(t), \boldsymbol{I_1}(t), \boldsymbol{I_2}(t)\right)$, where each of the components is a J-dimensional vector, e.g., $\boldsymbol{S}(t) = \{S_j(t); j = 1, \dots, J\}$. The evolution of this state vector is then approximated by the below system of difference equations:

$$\begin{aligned}
S_j(t_n) &= S_j(t_{n-1})\left(1 - \lambda_j(t_{n-1})\delta t\right), \\
E_{1,j}(t_n) &= E_{1,j}(t_{n-1})(1 - \sigma\delta t) + S_j(t_{n-1})\lambda_j(t_{n-1})\delta t, \\
E_{2,j}(t_n) &= E_{2,j}(t_{n-1})(1 - \sigma\delta t) + E_{1,j}(t_{n-1})\sigma\delta t, \\
I_{1,j}(t_n) &= I_{1,j}(t_{n-1})(1 - \gamma\delta t) + E_{2,j}(t_{n-1})\sigma\delta t, \\
I_{2,j}(t_n) &= I_{2,j}(t_{n-1})(1 - \gamma\delta t) + I_{1,j}(t_{n-1})\gamma\delta t.
\end{aligned} \tag{S1}$$

where σ and γ are related to the mean durations of latent and infectious infection, d_L and d_I, via

$$\sigma = 2/d_L, \gamma = 2/d_I,$$

the infection hazard, $\lambda_j(t_{n-1})$, is found through the Reed-Frost formulation

$$\lambda_j(t_n) = 1 - \prod_{i=1}^{J} \left[(1 - \beta_{j,i}(t_n))^{I_{1,i}(t_n) + I_{2,i}(t_n)}\right]$$

where $\beta_{j,i}(t_n)$ is the $(j,i)^{\text{th}}$ entry of the time-varying $(A \times A)$ matrix $\boldsymbol{\beta}(t)$, the entries of which give the infection rate of a susceptible individual in stratum j due to a single infectious individual in stratum i. We relate this infection rate matrix to the epidemic's reproductive number, R_0, through

$$\boldsymbol{\beta}(t) = \mathbf{M}(t)\, {}^{\mathrm{R}_0}\!/_{R_0^*} \tag{S2}$$

where $\boldsymbol{M}(t)$ is a contact matrix describing the relative rates of contact between any two individuals of different strata, and R_0^* is the dominant eigenvalue of the next generation matrix $\boldsymbol{M}^*$ which has entries $M_{j,i}^* = N_j M_{j,i}(0) d_I$, where N_j is the size of the population in stratum j.

There are two keys output time series from the model. The first is the seropositivity, which is related to the levels of cumulative incidence in the population. This can be written

$$q_j^{\text{sero}}(t_n) = 1 - \frac{S_j(t_n)}{N_j}.$$

The second is the number of new infections. Within stratum, *j*, in the *n*th interval, these are denoted

$$\Delta_j^{(\text{infec})}(t_n) = S_j(t_{n-1})\lambda_j(t_{n-1})\delta t. \qquad \text{(S3)}$$

These are the outputs of the transmission model that act as inputs into the disease reporting model. The disease reporting model has as its outputs, time series composed of the expected number of reported healthcare events (here, either a GP consultation or a virological confirmation of illness). These are obtained through a convolution of a fraction of the incident infections,

$$\mu_j^{(\text{event})}(t_n) = \theta p_j^{(\text{event})}(t_n) \sum_{k=0}^{n} f_j(k; \mu_{\text{event}}, \sigma_{\text{event}}^2) \Delta_j^{(\text{infec})}(t_{n-k}) \qquad \text{(S4)}$$

where θ represents the proportion of infections that lead to febrile symptoms, $p_j^{(\text{event})}(t_n)$ is the, possibly time-varying, proportion of symptomatic cases that lead to a particular healthcare event, and $f_j(k; \mu_{\text{event}}, \sigma_{\text{event}}^2)$ is the probability that, assuming that all infections and disease reporting occur at the same point in each time interval, the time from the infection to the reporting of the healthcare event is $k\delta t$. It is then assumed that the observed number of events has a probability distribution that is centred on $\mu_j^{(\text{event})}(t_n)$.

S1.2 Model extensions

In this study, the daily counts of virologically confirmed cases in the early part of the pandemic can be reasonably assumed to be distributed with mean as calculated by Equation (S4), whereas the primary care consultations are very different. These counts contain some significant contamination from individuals suffering non-pandemic influenza like illness (ILI) – they have the symptoms, but not the infection, in which we are interested. This level of 'background' infection is atypical of normal 'flu' seasons as there is a heightened sensitivity amongst the public to the presence of symptoms, and this sensitivity fluctuates rapidly due to, amongst other factors, the levels of media reporting prevalent at any time. So the existence of relevant historical data is unlikely. Furthermore, there is a strong within-week pattern in the reporting of primary care consultations. There is little to no reporting of consultations at weekends, whilst many delay their consultation until the following Monday (Supplementary Fig. S1). Therefore, when considering daily data, we need to consider a way to account for this pattern. Previously[1], these factors were inadequately handled and so a large degree of overdispersion was evident in the data, which had to be modelled using the negative binomial distribution. How the model has since been adapted to account for these two obscuring factors is outlined in the next two sub-sections, though we retain the negative binomial distribution in the calculation of the likelihood of the primary care consultation data.

S1.2.1 Background consultations

In Birrell *et al.*[1], posterior inference for the "background" consultation rates in London $\left(B_j(t)\right)$ was obtained via a log-linear generalised linear model (glm) in a two-stage process. The first stage generated priors for the parameters of the glm by applying a simple joint model for consultations and virological positivity

data to data from the English regions outside of London. In the second stage, posterior distributions were obtained by including the glm as part of the epidemic model. In the modelling of this paper, the data from within- and outside-London are unified as the models considered incorporate a spatial dimension. This spatial aspect allows the borrowing of strength across the regions and, as a result, there is sufficient information to estimate background parameters without the requirement for external information or modelling to generate an informative prior. As in Birrell *et al*[1], within each stratum, the background rates of consultation are assumed to be piecewise constant, remaining constant over intervals that are approximately fortnightly in duration. If time is broken into K intervals, then, neglecting any spatial variation for the moment, the background consultation rate is given by the glm

$$\log\{B_j(t_n)\} = x_{t_n,j}^{\mathrm{T}} \boldsymbol{\beta}^{(B)}, n = 1, \ldots, T; i = 1, \ldots, A.$$

The precise nature of the design vectors $\boldsymbol{x}_{t_n,j}$ and hence the interpretation of the parameters $\boldsymbol{\beta}^{(B)}$ is discussed once we formally introduce the spatial aspect of our modelling in Supplementary Section S1.3.1.

Once the $B_j(t_n)$ have been calculated, define $\mu_j^*(t_n) = \mu_j^{(\mathrm{GP})}(t_n) + B_j(t_n)$, then the virological swabs taken within time interval $[t_{n-1}, t_n)$, in stratum j have positivity given by

$$q_j^{\mathrm{viro}}(t_n) = 1 - \frac{B_j(t_n)}{\mu_j^*(t_n)}.$$

S1.2.2 Day of the week reporting effects: A highly evident artefact in the daily number of reported primary care consultations is the within-week pattern of consultation (Supplementary Fig. S1). Doctor surgeries are rarely open on weekends and are even

less likely to report/swab patients on these days. Patients whose illness may fall on a weekend may delay their consultation until the following Monday. If we denote $d(t_n) = 1, \ldots, 7$ to be the day of the week on which the nth interval falls, then the day of the week effects upon reporting can be accounted for by introducing scalars c_d such that

$$\mu_j'(t_n) = c_{d(t_n)}\mu_j^*(t_n) \text{ and } \prod_{d=1}^{7} c_d = 1 \quad \text{(S5)}$$

The second equation in Supplementary Equation (S5) is enforced to ensure identifiable effects.

Despite the model adaptations listed above and therefore a much reduced degree of dispersion, the primary care consultations are still assumed to have negative binomial distribution with mean given by Supplementary Equation (S5) and variance $\mu_j'(t_n)(1 + \eta_{t_n})$, where $\eta_{t_n} > 0$ is a (possibly time-varying) dispersion factor.

S1.2.3 Multi-region modelling Epidemics of the type described by our SEEIIR model (Supplementary Equation (S1)) are most effective when the studied population can be stratified into groups within which transmission dynamics are homogeneous. There was significant regional heterogeneity in the 2009 A/H1N1pdm outbreak[2,3]. Initially, Greater London and West Midlands were the only two regions with a sustained spring/summer first wave of infection. If the pandemic was subsequently modelled at a national level, the model ignores this heterogeneity, a phenomena compounded by the fact that important virological swabbing data over-sampled the regions of high incidence, leading to an over-pessimistic view of the spread of infection.

It is important, therefore, to stratify by region, to the finest spatial level that our data will permit. Unfortunately, this stratification, in practical terms, is heavily constrained by the sample sizes of the virological and serological data.

To handle this regional variation, we stratify the transmission dynamics according to our region definitions. We consider two main approaches to account for spatial variation in infection. In an approach that we label the 'parallel-region' model, each region has its own epidemic and there is no inter-region transmission. The second approach, we term the 'meta-region' model, we presume that census commuter data can be used as a proxy for rates of contact between individuals of different regions.

These two modelling approaches will be discussed in detail in the next two subsections.

S1.3 Parallel-region model

S1.3.1 Parameterisation:

The model of Birrell *et al.*[1] is implemented in each of our four regions, each with no external influence upon transmission in the other regions. It differs in that many parameters are shared across the regions, whereas, for the background levels of consultation, the regional differences in consultation rates are modelled via log-linear regression. The transmission model is parameterised in terms of the initial rate of epidemic growth within each region, ψ_r, and a further region specific parameter, υ_r. These parameters are a reparameterisation of the region-specific reproductive number, $R_{0,r}$, obtained via the expression[4]

$$R_{0,r} = \psi_r d_I \frac{\left(\frac{\psi_r d_L}{2}+1\right)^2}{1-\frac{1}{\left(\frac{\psi_r d_I}{2}+1\right)^2}}, \qquad \text{(S6)}$$

and the size of the initial seed of infection $I_{0,r}$ through[1]

$$I_{0,r} = \frac{d_I e^{\upsilon_r} \sum_a N_a}{p_{r,1}^{(\mathrm{GP})}(t_0) R_{0,r}}, \tag{S7}$$

The region specific $R_{0,r}$ and $I_{0,r}$ ensure that different timings and epidemic growth rates are possible in each of the regions. Parameters that describe viral properties are typically held fixed over the regions but those that are more behavioural in nature are allowed to vary. Column 3 of Table 1 gives the type of spatial variation used for each of the parameter groups. SI 2.2.1 and 2.2.2 to Birrell *et al.*[1] describe two modelling challenges, the parameterisation of the contact matrices and the propensity of the ILI symptomatic to consult with a GP. These challenges are still relevant and, subject to a slight change in notation, are handled in an identical fashion within each region in the modelling presented here. The consultation propensity, $p^{(\mathrm{GP})}$ is now, however, region specific, so that there are now 32, not 8, parameters of this type and $p_{r,a}^{(\mathrm{GP})}(t_n)$ gives the value of the propensity in region r for individuals of age group a in the nth time interval.

The one parameter grouping where the spatial variation is modelled to any extent is the background consultation parameters. As discussed in Supplementary S1.2.1, the background consultation rates are constant over approximately fortnightly intervals. If we seek, therefore, to evaluate the background rate of GP consultation in region, r, in time interval, $[t_{n-1}, t_n)$ among age groups a, $B_{r,a}(t_n)$, we pick the fortnightly interval $\tau \stackrel{\mathrm{def}}{=} \tau(t_n) \in \{1, \ldots, T\}$ that contains the interval $[t_{n-1}, t_n)$. The overall model was implemented a number of times, initially starting with a saturated sub-model for $B_{r,a}(t_n)$:

$$\log\left(\boldsymbol{B}_{r,a}(\boldsymbol{t}_n)\right) = \boldsymbol{\mu} + \boldsymbol{\alpha}_r + \boldsymbol{\beta}_\tau + \boldsymbol{\gamma}_a + \boldsymbol{\delta}_{r\tau} + \boldsymbol{\epsilon}_{ra} + \boldsymbol{\varsigma}_{\tau a} + \boldsymbol{\tau}_{r\tau a};$$

$$r \in \{L, W, N, S\}, \tau = 1, \dots, T_X, a = 1, \dots, A.$$

$$\log\left(\boldsymbol{B}_{\boldsymbol{r},\boldsymbol{a}}(\boldsymbol{t}_{\boldsymbol{n}})\right) = \boldsymbol{\mu}^* + \boldsymbol{\alpha}_{\boldsymbol{r}}^* + \boldsymbol{\beta}_{\tau}^* + \boldsymbol{\gamma}_{\boldsymbol{a}}^* + \boldsymbol{\delta}_{\boldsymbol{r}\tau}^* + \boldsymbol{\epsilon}_{\boldsymbol{ra}}^* + \boldsymbol{\varsigma}_{\tau\boldsymbol{a}}^* + \boldsymbol{\tau}_{\boldsymbol{r}\tau\boldsymbol{a}}^*;$$

$$r \in \{L, W, N, S\};\ \tau = T_X + 1, \dots, T;\ a = 1, \dots, A.$$

Here T_x indicates the time interval that concluded at the same time as the launch of the National Pandemic Flu Service (NPFS) on July 23 2009, and the regions {L,W,N,S} correspond to London, West Midlands, North and South respectively. Note that this specification implies that there are separate and non-interacting models for the background consultation rate in the pre- and post-NPFS eras.

Using the parallel-region model, and carrying out a crude model selection on the basis of the posterior mean deviance, 2 log$\{L(\varphi)\}$ given in Supplementary S1.5 Inference, the model was repeatedly implemented with higher order terms systematically removed in the hope of finding a more parsimonious parameterisation without incurring any significant lack of fit to the data. Additionally, some age groups and regions were paired together, to cover gaps where data denominators were too small to warrant extra age/region effects. The final model settled upon, and the one that is applied over the course of the rest of the analysis in the manuscript, is:

$$\log\left(\boldsymbol{B}_{\boldsymbol{r},\boldsymbol{a}}(\boldsymbol{t}_{\boldsymbol{n}})\right) = \boldsymbol{\mu} + \boldsymbol{\alpha}_{\boldsymbol{r}} + \boldsymbol{\beta}_{\tau} + \boldsymbol{\gamma}_{\boldsymbol{a}} + \boldsymbol{\delta}_{\boldsymbol{r}\tau} + \boldsymbol{\epsilon}_{\boldsymbol{ra}}; r \in \{L, W, S\};\ \tau = 1, \dots T_X; a = 2, \dots, A$$

$$\log\left(\boldsymbol{B}_{\boldsymbol{r},\boldsymbol{a}}(\boldsymbol{t}_{\boldsymbol{n}})\right) = \boldsymbol{\mu}^* + \boldsymbol{\alpha}_{\boldsymbol{r}}^* + \boldsymbol{\beta}_{\tau}^* + \boldsymbol{\gamma}_{\boldsymbol{a}}^* + \boldsymbol{\epsilon}_{\boldsymbol{ra}}^*; r \in \{L, W, S\};\ \tau = T_X + 1, \dots, T; a = 2, \dots, A$$

with sum-to-zero constraints implemented on all regression terms and with $B_{N,a}(t_n) = B_{S,a}(t_n)$ and $B_{r,1}(t_n) = B_{r,2}(t_n)$. These last two constraints equate the background in the North and South (there is insufficient virological data in the North to accurately estimate the background here) and the rates in the < 1 age group are assumed to be the same as in the 1-4 age group (the QSurveillance data don't

distinguish between these age groups,[5] only the virological data do,[6] so there is a lack of information to separately estimate the two).

S1.3.2 Mixing As there is no inter-regional transmission there is a separate system of forward simulation Equations (1) for each region. The pattern of transmission is determined by the $(A \times A)$ contact matrices $\mathbf{M}(t)$, that are based on contact rates derived from POLYMOD data from the United Kingdom[7]. This allows for a significant computational speed-up as the calculation of the likelihood of the data in each region can utilise parallel computation.

S1.3.3 Seeding Supplementary Equation (S7) gives the initial number of infectives in each region. Within these regions, these infectives are distributed across the age-defined strata according to the normalised eigenvector of the next generation matrix $\mathbf{M}^*$ corresponding to eigenvector R_0^* (see Supplementary Equation (S2)). The relation between the initial number of infectives and the numbers of individuals in each disease state at t_0 are determined from $I_{0,r}$, the initial rate of epidemic growth ψ_r and the mean infectious and latent periods. There are, therefore, two parameters per region that describe the initial condition of the epidemic, ψ_r and υ_r.

S1.4 Meta-region model

S1.4.1 Parameterisation In the meta-region approach, the four regions are linked by commuter flows. Together they can be regarded as one single region, the population within being divided into strata defined by both age group and region. In Equations (1) and (S1), the index j could be related to the age $a = 1, \ldots, A,$ and region $r = 1, \ldots, R$, by assigning strata to be $j = a + A(r - 1)$. This means that we have the same system of

equations as in each of the parallel region models, but the number of strata has increased such that $j \epsilon (1, \ldots, RA)$, where R and A are the total number of regions and age groups, respectively. This therefore leads to some fundamental differences between the parallel-region approach and the meta-region approach:

• In the parallel-region approach, each region has its own exponential growth rate ψ_r, while in the meta-region approach it is a global measure. By Supplementary Equation (S6), which still holds within the meta-regional model and for which a detailed proof is given in Supplementary Section 3, this holds also for regional reproductive numbers $R_{0,r}$.

• The initial seed of infection in the parallel-region approach consists of a number of infectious individuals in each region. Again, in the meta-region approach, this is a global value, I_0.

• In Equation (3) and Supplementary Equation (S2), the contact matrices $\mathbf{M}(t)$ are $(A \times A)$ matrices for the parallel-region model. Now they are $(RA \times RA)$ matrices $\Pi(t)$ (see equation (4)), a consequence of the now possible interactions between age groups in different regions.

S1.4.2 Density dependence and mixing among regions The last point above requires some thought as to how the contact matrices are to be specified. Supplementary Table S1 gives commuting matrices $\mathbf{C}(a)$ for each of the four adult age groups describing the proportion of individuals from each age group who move to other regions (or stay in their native region) over the course of a specified day. We assume that individuals younger than 16 years do not commute. Let's presume that these commuter movements cover a fraction of a commuter's total time ξ. In this proportion of time, transmission is only possible between two individuals if they are in the same region. Assuming that

commuting movements all take place at the same time, an individual native to region r of age group a will be in the same region as an individual native to region s of age group b with probability $\sum_{v=1}^{R} C_{rv}(a)C_{sv}(b)$. We assume that, once in a region, contacts between any two individuals will occur in proportion to POLYMOD-estimated rates of contact between members of their respective age groups.[7] Further, it is assumed that there is some density dependent effects on transmission (i.e. contacts are more likely between any two individuals when they are in a lower-populated region). Based on these assumptions, we have a contact matrix Π giving rates of contact between individuals of stratum (r, a) indexed by $j = a + A(r-1)$ and stratum (s, b) indexed by $i\big(= b + A(s-1)\big)$ to be as defined in Equation (3), where the 'daytime' populations for each stratum are given by $N_{v,a}^{\mathrm{D}} = \sum_{s=1}^{R} C_{sv}(a)N_{s,a}$.

For the model fitting in this paper, we use a value for ξ that assumes that individuals commute for a half-day period, on five days out of seven in each week, so we set $\xi = (1/2) \times (5/7) = 5/14$. Transmission in this meta-region model, therefore, is directly characterised by the interactions between regions arising from commuter flux as well as within-region interactions. This is the approach of Keeling *et al.*,[8] though alternatives exist in the methods of Eggo *et al.*[9] and Gog *et al.*[10], where short-term migrations are modelled via a diffusion process, e.g. the gravity model.

The denominators of Equation (3) give the prevalent degree of density dependence. There are two things to consider here:

- The value of α. A value of $\alpha = 0$ corresponds to frequency dependent transmission (two people are equally like to interact regardless of the population

size of their region), whereas $\alpha = 1$ corresponds to a density dependent effect upon transmission. Values of $\alpha = 0$, 0.5, and 1 will be considered.

- How density dependence should be incorporated. Equation (3) uses dependence upon the population size of the strata of the uninfected individual in an infectious contact. However, it is not obvious why it should be this and not the population size of the region to which the infectious individual belongs, and, furthermore, in the parallel-region model it is assumed that there are no density dependent effects across the age groups. Therefore, as an alternative, it is proposed to replace $N_{v,a}^{\mathrm{D}}$ with the region-wide population size $N_v^{\mathrm{D}} = \sum_{a=1}^{A} N_{v,a}^{\mathrm{D}}$ and $N_{v,a}^{\mathrm{N}}$ with $N_v^{\mathrm{N}} = \sum_{a=1}^{A} N_{v,a}^{\mathrm{N}}$. This tests whether it is the population size of a region that is important in density dependence, as opposed to the age-group constitution of that population.

S1.4.3 Seeding The POLYMOD-based contact matrices used in the parallel-region model lead to very rapid mixing and the disease-free equilibrium of infection is attained after only a relatively short time. Therefore, fitted epidemic curves are not particularly sensitive to the choice of the initial seeding of infection across the strata.

On the contrary, the spatial heterogeneity in population sizes and patterns of movements in the meta-region model means that the initial seeding of infection will affect the geographical spreading of the predicted epidemics.[11, 12] As illustrated in the main text, the expanded ($RA \times RA$) contact matrix Π in the meta-region model has a near-block diagonal structure that leads to very slow mixing. This renders doubtful the validity of using an asymptotically-justified seeding as the disease-free equilibrium is

attained very slowly (if at all). Therefore, in addition to estimating the number of initial infections, and unlike in the parallel-region model where the distribution of this seeding among the seven age groups within each region is determined by the dominant eigenvalue of the next-generation matrix, the initial distribution of infection has to be estimated, or, as here, some practical alternatives for specifying the distribution need to be formulated. We consider: the next-generation matrix-based seeding and label this the 'nextgen' approach; an 'empirical' seeding, representing the distribution of an initial number of confirmed cases observed prior to 1st May, 2009 which should hopefully be indicative of the early pattern of infection; and an 'extended empirical' seeding, a hybrid of the two previous approaches, where the empirical seeding is used to divide the initial infective individuals among the regions, and within each region, the dominant eigenvalue of the within-region contact matrix is used to determine the distribution across the age groups - the distribution of the seeds that would be used were this region to be considered in isolation, as in the parallel-region model and in Birrell *et al.*[1]

S1.4.4 Random Commuters vs. Fixed Commuters Individuals within each stratum are assumed to be homogeneous of behaviour. That is, in any given interval, any individual within a specific stratum is equally likely to be one who commutes. This may well prove to be a gross simplification because:

- The bulk of commuting is done by a much smaller sub-population within a stratum, each of whom commutes on a regular basis.
- Infectious individuals are still assumed to carry on with normal commuting behaviour, whereas in reality, they may well be too ill to travel.

The first simplification we term to be an assumption of 'commuting at random'. To consider the possibility that there is a fixed sub-population within each stratum who

commute regularly, we further stratify the adult age-groups into 'commuters' and 'non-commuters'. Commuters are assumed not to stay in their native region during working hours, whereas non-commuters stay in their native region with certainty. So, rather than RA strata (in our example, this is $7\times4 = 28$), we partition further. Four of the seven age groups are of adult-age individuals and can be further divided into commuters and non-commuters classes. Therefore we now consider $(A + 4)\times R = 44$ strata. In this example, the expanded matrix has an identical mathematical expression as before (Equation (3), *main text*), once $C_{rs}(a)$ is replaced by $C^*_{rs}(a)$, where

$$C^*_{rr}(a) = \begin{cases} 1, & a \text{ belongs to a non-commuter class} \\ 0, & a \text{ belongs to a commuter class} \end{cases}$$

$$C^*_{rs}(a) = \begin{cases} 0, & a \text{ belongs to a non-commuter class} \\ \dfrac{C_{rs}(a)}{1 - C_{rr}(a)}, & a \text{ belongs to a commuter class} \end{cases}$$

The assumption of commuting at random speeds up the spread of infection across the four regions while the fixed commuting assumption increases the transiency of any commuting effects and results in greater heterogeneity in the times of peak infection across the regions. However, simulations have shown that the peak size and attack rate are insensitive to the commuting assumption. We shall investigate whether the 'fixed commuters' assumption can improve the model fit.

S1.5 Inference

The statistical model developed was implemented within the Bayesian framework. Here, parameter inference is based upon posterior probability distributions derived for the vector of parameters through the combination of the likelihood of the data and pre-specified prior distributions for the parameter components. Posterior distributions are not typically available in an easy-to-express analytic form. Instead, they have to be

approximated through sampling algorithms. Here Markov Chain Monte Carlo (MCMC) has been used, implemented using bespoke C++ code.

Having obtained approximations to the posterior distributions, inference can be made about other epidemic quantities of interest, where the quantity can be expressed as a function of our parameters (such as the epidemic trajectory, epidemic attack rate etc.). The Bayesian framework specifies how the uncertainty about parameters that is encapsulated in their posterior distributions is propagated to functions of the parameter vector, resulting in posterior distributions for the quantities of interest.

S1.5.1 Likelihood The likelihood function is merely an extension of that which is expressed in Birrell *et al.*[1] If we denote the collection of all the model parameters by the vector $\boldsymbol{\varphi}$, and

i. w_{rna} is a realisation of W_{rna}, the random variable representing the number of positive results in a sample of m^{v}_{rna} virologically tested swabs taken in region r in the nth interval, $[t_{n-1}, t_n)$ from individuals in age group a. This data is assumed to have binomial distribution.

ii. x_{rna} is a realisation of X_{rna}, the random variable giving the number of reported lab-confirmed cases in the interval $[t_{n-1}, t_n)$ in region r, amongst individuals in age group a. This data is assumed to be Poisson distributed.

iii. y_{rna} is a realisation of Y_{rna}, the random variable giving the number of GP consultations in the interval $[t_{n-1}, t_n)$ in region r, amongst individuals in age group a. This data is assumed to have a negative-binomial distribution.

iv. z_{rna} is a realisation of Z_{rna}, the random variable giving, in a test for the presence of immunity-conferring A/H1N1pdm antibodies, the number of

positive results from m^s_{rna} blood sera samples taken in the interval $[t_{n-1}, t_n)$ in region r, from individuals in age group a. This data is assumed to have binomial distribution.

Conditional upon the model parameters, it is assumed that all observations are independent. Therefore, the likelihood can simply be written:

$$L(\boldsymbol{\varphi}) = \prod_{r=1}^{R} \prod_{n=1}^{K} \prod_{a=1}^{A} \{L(w_{rna} | m^v_{rna}, \boldsymbol{\varphi}) \times L(x_{rna} | \boldsymbol{\varphi}) \times L(y_{rna} | \boldsymbol{\varphi}) \times L(z_{rna} | m^S_{rna}, \boldsymbol{\varphi})\}.$$

It is worth noting how the (region-specific) overdispersion parameter for the GP consultation data, η_{t_n} helps to form the likelihood. The negative-binomial distribution has mean $\mu'_{ra}(t_n)$, analogous to the single region quantity expressed in Supplementary Equation (S5), and variance given by $\mu'_{ra}(t_n)(1 + \eta_{r,t_n})$. In this parameterisation, the negative binomial likelihood is:

$$L(y_{rna} | \varphi) = \frac{\Gamma(y_{rna} + r_{rna})}{\Gamma(r_{rna})\Gamma(y_{rna} + 1)} \left(\frac{1}{1 + \eta_{r,t_n}}\right)^{r_{rna}} \left(\frac{\eta_{r,t_n}}{1 + \eta_{r,t_n}}\right)^{y_{rna}},$$

where $r_{rna} = \mu'_{ra}(t_n)/\eta_{r,t_n}$. The time dependence in the dispersion parameter exists through a single change point at the time of the NPFS launch, reflecting a systematic change in GP consultation behaviour of the population.

S1.5.2 Priors Supplementary Table S2 provides a summary list of all the model parameters. Some are free parameters for which priors need to be specified, to enable their estimation (in the form of posterior distributions), and others are fixed parameters, assumed to be known. SI2.3.2 to Birrell *et al.*[1] details the justification behind many of the prior distributions listed in the table. Here, some parameters have an added dependency on the index r, indicating regional variation. In this case, parameters are independently and identically distributed across regions. There are two

additional rows to the table corresponding to the parameters of the background consultation rate and the day of the week reporting effects. Both of these sets of parameters have multivariate normal prior distributions, of dimension 61 and 6 respectively. The covariance matrices $\mathbf{V}^{(B)}$ and $\mathbf{V}^{(c)}$ are designed such that the rates of background consultation, $B_{a,r}(t_n)$, and the within-week reporting effects, c_d, are, *a priori*, everywhere identically distributed.

S2. Further Results

Overall, our comparisons suggest that the choice of $\alpha = 1$, and the use of N_r rather than $N_{r,a}$ in Equation (3) give a better model fitting as shown in Supplementary Table S3. This points to a density-dependent effect upon transmission, but makes the somewhat sensible choice that the density dependence is governed by the overall population size, and not the size of specific strata. Of the seeding options, the empirically based seeds performed most strongly, with the *extended-empirical* being the preferred choice. The distinction between commuters and non-commuters in the stratification typically led to a mild improvement in fit under most parameterisations. However, when considering a 'best' model, i.e. with $\alpha = 1$, regional density dependent effects and the *extended empirical* seed, the improvement in the expansion to 44 strata is no longer evident. As this model takes substantially longer to implement, the reduced stratification that does not distinguish between commuters and non-commuters is preferred.

S2.1 The Estimated Epidemic and Goodness-of-fit

Figure 2 gives the temporal pattern of infections under both modelling approaches by both age-group and region. Supplementary Table S4 additionally provides posterior

means for the overall age-specific attack rates by both age-group and region, and partitions this among the first and second waves of infection. Fig. 2 presents qualitatively similar temporal patterns in each of the four regions. For example, the four regions each attained two peaks triggered by the start of a school holiday in weeks 29 and 43, with the exception of the West Midlands which experienced a later second peak in week 49. The occurrence of this later peak in the West Midlands could, perversely, be attributable to the fact that a greater portion of its supply of susceptibles is drained by the first wave, leading to very slow second wave growth that manages to persist over the short autumn holiday period, whereas the epidemic has 'burnt out' in the other regions by this time. The two models demonstrate, furthermore, that the temporal patterns in the North and South are very much dominated by the second wave of infections, whereas the peak of infection for London and West Midlands lie in the first wave (even if their second waves may have a greater cumulative infection). Despite the delayed second peak, the West Midlands still has the lowest attack rate during the second wave.

There are two main differences in the results obtained under the two modelling approaches. First, there is a shift towards second wave infection in the meta-region model for London (demonstrably so from Fig. 2) and the North who have higher second-wave attack rates, whereas under the parallel-region model, the attack rate is higher in the first wave for these two regions, the opposite being true for West Midlands and the South. As expected, the age groups with the highest rates of incidence are the 5-14 year-olds (Supplementary Table S4), with attack rates over two waves centred upon 53% (58%), 53% (52%), 63% (62%), 63% (61%) in London, West Midlands, the North and the South respectively, estimated under the parallel-region (and meta-region)

model. The goodness of fit to the GP consultation data under both the parallel-region and meta-region models (as presented in the *main text*) are presented in Supplementary Figs. S2 and S3 respectively. The corresponding seropositivity and virological positivity plots are showcased in Supplementary Figs. S4 and S5. To the eye, all demonstrate a more than adequate fit to each of the datasets, with the fit to the parallel-region model seeming to be slightly superior (as evidenced from the posterior mean deviance statistics), particularly so for the GP consultation data where the observations fall within the (rather tight) 95% credible intervals in a significantly higher proportion of cases.

S3. The relationship between the exponential growth rate (ψ_r) and the reproductive number (R_0) in a heterogeneously-mixing population

The mathematical induction method was applied to prove the relationship given in Supplementary Equation (S6), i.e.

$$R_0 = \psi_r d_I \frac{\left(\frac{\psi_r d_L}{2} + 1\right)^2}{1 - \left(\frac{\psi_r d_I}{2} + 1\right)^{-2}} = d_I \frac{(\sigma + \psi_r)^2 (\gamma + \psi_r)^2}{(2\gamma + \psi_r)\sigma^2}, \tag{S8}$$

holds within a heterogeneously-mixing population. Here $d_I (= 2/\gamma)$ and $d_L (= 2/\sigma)$ are the expected infectious and latent periods, respectively.

We first prove Supplementary Equation (S8) holds when the population is composed of one stratum ($J = 1$) so that mixing is homogeneous. We need to prove that Supplementary Equation (S8) will hold for a population which is composed of $J = n+1$ strata provided it holds for a population of $J = n$ strata. For convenience, we write the epidemic system using the set of differential equations that the system of difference equations in Equation (1) seek to approximate; also we approximate the Reed-Frost

formulation of the force of infection used in our model specification (Equation (2)) is approximated by the mass action law.

1) J=1. A population is assumed to consist of only one stratum, and the SEEIIR epidemic is described by,

$$\frac{dS(t)}{dt} = -\lambda(t)S(t),$$
$$\frac{dE_1(t)}{dt} = \lambda(t)S(t) - \sigma E_1(t),$$
$$\frac{dE_2(t)}{dt} = \sigma E_1(t) - \sigma E_2(t),$$
$$\frac{dI_1(t)}{dt} = \sigma E_2(t) - \gamma I_1(t),$$
$$\frac{dI_2(t)}{dt} = \gamma I_1(t) - \gamma I_2(t),$$

With force of infection

$$\lambda(t) = 1 - [1 - \beta(t)]^{I_1(t)+I_2(t)} \approx \beta(t)[I_1(t) + I_2(t)],$$

where the infection rate $\beta(t)$ is related to the contact rate $\Pi(t)$ through

$$\beta(t) = \Pi(t)\frac{R_0}{N\Pi(0)d_I}.$$

In particular,

$$\beta(0) = \frac{R_0}{Nd_I}.$$

The equations governing the rate of change in the numbers within each infectious state can be written:

$$\frac{d}{dt}\begin{pmatrix} E_1 \\ E_2 \\ I_1 \\ I_2 \end{pmatrix} = \begin{pmatrix} -\sigma & 0 & \bar{\beta}(t) & \bar{\beta}(t) \\ \sigma & -\sigma & 0 & 0 \\ 0 & \sigma & -\gamma & 0 \\ 0 & 0 & \gamma & -\gamma \end{pmatrix}\begin{pmatrix} E_1 \\ E_2 \\ I_1 \\ I_2 \end{pmatrix} = \mathbf{\Theta}_1(t)\begin{pmatrix} E_1 \\ E_2 \\ I_1 \\ I_2 \end{pmatrix}.$$

Here $\bar{\beta}(t) \equiv \beta(t)S(t)$. Assume that the initial infections increase exponentially at a growth rate ψ_r, we have $\mathbf{\Theta}_1 \boldsymbol{v}_1(\mathrm{t}) = \psi_r \boldsymbol{v}_1(\mathrm{t})$, with $\boldsymbol{v}_1(t) = (E_1(t), E_2(t), I_1(t), I_2(t))^T$. Hence we can identify the characteristic equation at time $t = 0$:

$$|\mathbf{\Theta}_1 - \psi_r \mathbf{I}_4| = \begin{vmatrix} -\sigma - \psi_r & 0 & \bar{\beta}(0) & \bar{\beta}(0) \\ \sigma & -\sigma - \psi_r & 0 & 0 \\ 0 & \sigma & -\gamma - \psi_r & 0 \\ 0 & 0 & \gamma & -\gamma - \psi_r \end{vmatrix} = a - b\bar{\beta}(0).$$

Here $a \equiv (\sigma + \psi_r)^2 (\gamma + \psi_r)^2$ and $b \equiv \sigma^2 (2\gamma + \psi_r)$. In the above expression, I_4 represents the identity matrix of dimension 4. We notice that

$$\Lambda \equiv \frac{a}{b} = \frac{(\sigma + \psi_r)^2 (\gamma + \psi_r)^2}{(2\gamma + \psi_r)\sigma^2} \tag{S9}$$

is the eigenvalue of the contact matrix $\bar{\beta}(0)$ (trivially so as this is a scalar quantity). The next generation matrix (and its eigenvalue) at time $t=0$ is $\bar{\beta}(0) d_I = \Lambda d_I = R_0$, as given in Supplementary Equation (S8).

2) Assume that a population is divided into $J = n$ strata and the SEEIIR epidemic is described by, for stratum j,

$$\begin{aligned} \frac{dS_j(t)}{dt} &= -\lambda_j(t) S_j(t), \\ \frac{dE_{1,j}(t)}{dt} &= \lambda_j(t) S_j(t) - \sigma E_{1,j}(t), \\ \frac{dE_{2,j}(t)}{dt} &= \sigma E_{1,j}(t) - \sigma E_{2,j}(t), \\ \frac{dI_{1,j}(t)}{dt} &= \sigma E_{2,j}(t) - \gamma I_{1,j}(t), \\ \frac{dI_{2,j}(t)}{dt} &= \gamma I_{1,j}(t) - \gamma I_{2,j}(t), \end{aligned} \tag{S10}$$

The force of infection acting upon stratum j is

$$\lambda_j(t) = \textstyle\sum_{i=1}^{n} \beta_{j,i}(t) \left[I_{1,i}(t) + I_{2,i}(t) \right]. \tag{S11}$$

Here $\beta_{j,i}(t)$ is the infection rate of a susceptible individual in stratum *j* due to a single infectious individual in stratum *i*. It is related to the $(j,i)^{\text{th}}$ entry of the contact matrix $\boldsymbol{\Pi}(t)$ which characterises the mixing rate at time t through

$$\beta_{j,i}(t) = \Pi_{j,i}(t)\frac{R_0}{R_0^*}$$

Here R_0^* is the dominant eigenvalue of the next generation matrix $\boldsymbol{\Pi}^*$ which has entries $\Pi^*_{j,i} = N_j \Pi_{j,i}(0) d_I$, where N_j is the size of the population in stratum *j*.

Equations governing the changing numbers within each infectious state can be written as

$$\frac{d}{dt}\boldsymbol{v}_n(t) = \boldsymbol{\Theta}_n(t)\boldsymbol{v}_n(t) \tag{S12}$$

Here $\boldsymbol{v}_n(t) = \left(E_{1,1}(t), E_{2,1}(t), I_{1,1}(t), I_{2,1}(t), E_{1,2}(t), \dots, I_{2,n}(t)\right)^T$ and

$$\boldsymbol{\Theta}_n(t) = \begin{pmatrix} \Lambda_{1,1}(t) & \Lambda_{1,2}(t) & \dots & \Lambda_{1,n}(t) \\ \Lambda_{2,1}(t) & \Lambda_{2,2}(t) & \dots & \Lambda_{2,n}(t) \\ \dots & \dots & \dots & \dots \\ \Lambda_{n,1}(t) & \Lambda_{n,2}(t) & \dots & \Lambda_{n,n}(t) \end{pmatrix} \tag{S13}$$

Within the above expression, the block matrices are

$$\Lambda_{j,j}(t) \equiv \begin{pmatrix} -\sigma & 0 & \beta_{j,j}(t)S_j(t) & \beta_{j,j}(t)S_j(t) \\ \sigma & -\sigma & 0 & 0 \\ 0 & \sigma & -\gamma & 0 \\ 0 & 0 & \gamma & -\gamma \end{pmatrix} = \begin{pmatrix} -\sigma & 0 & \bar{\beta}_{j,j}(t) & \bar{\beta}_{j,j}(t) \\ \sigma & -\sigma & 0 & 0 \\ 0 & \sigma & -\gamma & 0 \\ 0 & 0 & \gamma & -\gamma \end{pmatrix}$$

and

$$\Lambda_{j,i}(t) \equiv \begin{pmatrix} 0 & 0 & \beta_{j,i}(t)S_j(t) & \beta_{j,i}(t)S_j(t) \\ 0 & 0 & 0 & 0 \\ 0 & 0 & 0 & 0 \\ 0 & 0 & 0 & 0 \end{pmatrix} = \begin{pmatrix} 0 & 0 & \bar{\beta}_{j,i}(t) & \bar{\beta}_{j,i}(t) \\ 0 & 0 & 0 & 0 \\ 0 & 0 & 0 & 0 \\ 0 & 0 & 0 & 0 \end{pmatrix},$$

where $i, j \in \{1,2,\dots,n\}$ and $i \neq j$. Letting $\bar{\beta}_{j,i}(t) \equiv \beta_{j,i}(t)S_j(t)$, we define the matrix

$$\bar{\boldsymbol{\beta}}_n(t) = \begin{pmatrix} \bar{\beta}_{1,1}(t) & \bar{\beta}_{1,2}(t) & \dots & \bar{\beta}_{1,n}(t) \\ \bar{\beta}_{2,1}(t) & \bar{\beta}_{2,2}(t) & \dots & \bar{\beta}_{2,n}(t) \\ \dots & \dots & \dots & \dots \\ \bar{\beta}_{n,1}(t) & \bar{\beta}_{n,2}(t) & \dots & \bar{\beta}_{n,n}(t) \end{pmatrix} \tag{S14}$$

Assuming the initial infections increase exponentially at growth rate ψ_r, we have

$$\mathbf{\Theta}_n(t)\boldsymbol{v}_n(t) = \psi_r \boldsymbol{v}_n(t),$$

giving rise to the characteristic equation at time $t = 0$

$$|\mathbf{\Theta}_n(t) - \psi_r \mathbf{I}_{4n}| = \begin{vmatrix} \Lambda_{1,1}(t) - \psi_r \mathbf{I}_4 & \Lambda_{1,2}(t) & \cdots & \Lambda_{1,n}(t) \\ \Lambda_{2,1}(t) & \Lambda_{2,2}(t) - \psi_r \mathbf{I}_4 & \cdots & \Lambda_{2,n}(t) \\ \cdots & \cdots & \cdots & \cdots \\ \Lambda_{n,1}(t) & \Lambda_{n,2}(t) & \cdots & \Lambda_{n,n}(t) - \psi_r \mathbf{I}_4 \end{vmatrix}$$

This can be reduced to

$$|\mathbf{\Theta}_n(t) - \psi_r \mathbf{I}_{4n}| = (-1)^n \begin{vmatrix} b\bar{\beta}_{1,1}(0) - a & b\bar{\beta}_{1,2}(0) & \cdots & b\bar{\beta}_{1,n}(0) \\ b\bar{\beta}_{2,1}(0) & b\bar{\beta}_{2,2}(0) - a & \cdots & b\bar{\beta}_{2,n}(0) \\ \cdots & \cdots & \cdots & \cdots \\ b\bar{\beta}_{n,1}(0) & b\bar{\beta}_{n,2}(0) & \cdots & b\bar{\beta}_{n,n}(0) - a \end{vmatrix}$$

$$= (-1)^n b^n |\bar{\boldsymbol{\beta}}_n(0) - \Lambda \mathbf{I}_n|$$

$$= 0,$$

where $\Lambda = a/b$. This suggests expression (S9) as an eigenvalue of the matrix $\bar{\boldsymbol{\beta}}_n(0)$, as shown in (S14). The dominant eigenvalue of the next generation matrix $\Pi^* = \bar{\boldsymbol{\beta}}_n(0)d_I$ is then Λd_I as in Supplementary Equation (S8).

3) We want to prove that this relationship still holds for the population of $k = n+1$ strata. Again, we have:

$$\frac{d}{dt}\boldsymbol{v}_{n+1}(t) = \mathbf{\Theta}_{n+1}(t)\boldsymbol{v}_{n+1}(t)$$

Here $\boldsymbol{v}_{n+1}(t) = \left(\boldsymbol{v}_n(t), E_{1,n+1}(t), E_{2,n+1}(t), I_{1,n+1}(t), I_{2,n+1}(t)\right)^T$ and

$$\mathbf{\Theta}_{n+1}(t) = \left(\begin{array}{cccc|c} & & & & \mathbf{\Lambda}_{1,n+1}(t) \\ & \mathbf{\Theta}_n(t) & & & \mathbf{\Lambda}_{2,n+1}(t) \\ & & & & \vdots \\ & & & & \mathbf{\Lambda}_{n,n+1}(t) \\ \hline \mathbf{\Lambda}_{n+1,1}(t) & \mathbf{\Lambda}_{n+1,2}(t) & \cdots & \mathbf{\Lambda}_{n+1,n}(t) & \mathbf{\Lambda}_{n+1,n+1}(t) \end{array}\right)$$

Using the assumption for the case of $J = n$ strata, its characteristic equation at $t = 0$ (suppressing for ease of presentation any dependence on time) is:

$$\left|\boldsymbol{\Theta}_{n+1} - \psi_r \mathbf{I}_{4(n+1)}\right| = \left(\gamma + \psi_r\right)^2 \left(\sigma + \psi_r\right)^2 \left|\boldsymbol{\Theta}_n - \psi_r \mathbf{I}_{4n}\right| - \left(2\gamma + \psi_r\right)\sigma^2 \mathbf{A}_n = 0,$$

where,

$$\mathbf{A}_n = \begin{vmatrix} \Lambda_{1,1} - \psi_r \mathbf{I}_4 & \cdots & \Lambda_{1,n} & \widehat{\boldsymbol{\beta}}_{1,n+1} \\ \vdots & \ddots & \vdots & \vdots \\ \Lambda_{n,1} & \cdots & \Lambda_{n,n} - \psi_r \mathbf{I}_4 & \widehat{\boldsymbol{\beta}}_{n,n+1} \\ \widetilde{\boldsymbol{\beta}}_{n+1,1} & \cdots & \widetilde{\boldsymbol{\beta}}_{n+1,n} & \bar{\beta}_{n+1,n+1} \end{vmatrix}$$

The block matrices $\Lambda_{j,i} \equiv \Lambda_{j,i}(t)$ are as defined previously and the vectors are:

$$\widetilde{\boldsymbol{\beta}}_{n+1,i} \equiv \widetilde{\boldsymbol{\beta}}_{n+1,i}(t) = \begin{pmatrix} 0 & 0 & \bar{\beta}_{n+1,i}(t) & \bar{\beta}_{n+1,i}(t) \end{pmatrix}, \;\; i = 1, \dots, n$$

and

$$\widehat{\boldsymbol{\beta}}_{i,n+1} \equiv \widehat{\boldsymbol{\beta}}_{i,n+1}(t) = \begin{pmatrix} \bar{\beta}_{i,n+1}(t) \\ 0 \\ 0 \\ 0 \end{pmatrix}.$$

This can be broken down as

$$\mathbf{A}_n = (-1)^{n+2}\bar{\beta}_{1,n+1} \begin{vmatrix} \sigma & -\sigma-\psi_r & 0 & 0 & \cdots & 0 & 0 & 0 & 0 \\ 0 & \sigma & -\gamma-\psi_r & 0 & \cdots & 0 & 0 & 0 & 0 \\ 0 & 0 & \gamma & -\gamma-\psi_r & \cdots & 0 & 0 & 0 & 0 \\ \vdots & \vdots & \vdots & \vdots & \ddots & \vdots & \vdots & \vdots & \vdots \\ 0 & 0 & \bar{\beta}_{n,1} & \bar{\beta}_{n,1} & \cdots & -\sigma-\psi_r & 0 & \bar{\beta}_{n,n} & \bar{\beta}_{n,n} \\ 0 & 0 & 0 & 0 & \cdots & \sigma & -\sigma-\psi_r & 0 & 0 \\ 0 & 0 & 0 & 0 & \cdots & 0 & \sigma & -\gamma-\psi_r & 0 \\ 0 & 0 & 0 & 0 & \cdots & 0 & 0 & \gamma & -\gamma-\psi_r \\ 0 & 0 & \bar{\beta}_{n+1,1} & \bar{\beta}_{n+1,1} & \cdots & 0 & 0 & \bar{\beta}_{n+1,n} & \bar{\beta}_{n+1,n} \end{vmatrix}$$

$$+ \cdots + (-1)^{2n+1}\bar{\beta}_{n,n+1} \begin{vmatrix} -\sigma-\psi_r & 0 & \bar{\beta}_{1,1} & \bar{\beta}_{1,1} & \cdots & 0 & 0 & \bar{\beta}_{1,n} & \bar{\beta}_{1,n} \\ 0 & -\sigma-\psi_r & 0 & 0 & \cdots & 0 & 0 & 0 & 0 \\ 0 & 0 & -\gamma-\psi_r & 0 & \cdots & 0 & 0 & 0 & 0 \\ 0 & 0 & \gamma & -\gamma-\psi_r & \cdots & 0 & 0 & 0 & 0 \\ \vdots & \vdots & \vdots & \vdots & \ddots & \vdots & \vdots & \vdots & \vdots \\ 0 & 0 & 0 & 0 & \cdots & \sigma & -\sigma-\psi_r & 0 & 0 \\ 0 & 0 & 0 & 0 & \cdots & 0 & \sigma & -\gamma-\psi_r & 0 \\ 0 & 0 & 0 & 0 & \cdots & 0 & 0 & \gamma & -\gamma-\psi_r \\ 0 & 0 & \bar{\beta}_{n+1,1} & \bar{\beta}_{n+1,1} & \cdots & 0 & 0 & \bar{\beta}_{n+1,n} & \bar{\beta}_{n+1,n} \end{vmatrix}$$

$$+ \bar{\beta}_{n+1,n+1}\left|\boldsymbol{\Theta}_n - \psi_r \mathbf{I}_{4n}\right|.$$

After some algebraic operations we have

$$\mathbf{A}_n = (-1)^{n+2}\bar{\beta}_{1,n+1}\begin{vmatrix} b\bar{\beta}_{2,1} & b\bar{\beta}_{2,2}-a & \dots & b\bar{\beta}_{2,n} \\ \vdots & \vdots & \ddots & \vdots \\ b\bar{\beta}_{n,1} & b\bar{\beta}_{n,2} & \dots & b\bar{\beta}_{n,n}-a \\ b\bar{\beta}_{n+1,1} & b\bar{\beta}_{n+1,2} & \dots & b\bar{\beta}_{n+1,n} \end{vmatrix} + \cdots$$

$$-\bar{\beta}_{n,n+1}\begin{vmatrix} b\bar{\beta}_{1,1}-a & \dots & (2\gamma+\psi_r)\bar{\beta}_{1,n-1} & b\bar{\beta}_{1,n} \\ \vdots & \ddots & \vdots & \vdots \\ b\bar{\beta}_{n-1,1} & \dots & b\bar{\beta}_{n-1,n-1}-a & b\bar{\beta}_{n-1,n} \\ b\bar{\beta}_{n+1,1} & \dots & b\bar{\beta}_{n+1,n-1} & b\bar{\beta}_{n+1,n} \end{vmatrix}$$

$$+\bar{\beta}_{n+1,n+1}|\mathbf{\Theta}_n - \psi_r\mathbf{I}_{4n}|.$$

Putting this back, we have

$$|\mathbf{\Theta}_{n+1} - \psi_r\mathbf{I}_{4(n+1)}| = a|\mathbf{\Theta}_n - \psi_r\mathbf{I}_{4n}| - b\mathbf{A}_n$$

$$= \left(a - b\bar{\beta}_{n+1,n+1}\right)|\mathbf{\Theta}_n - \psi_r\mathbf{I}_{4n}|$$

$$+(-1)^{n+1}b\bar{\beta}_{1,n+1}\begin{vmatrix} b\bar{\beta}_{2,1} & b\bar{\beta}_{2,2}-a & \dots & b\bar{\beta}_{2,n} \\ \vdots & \vdots & \ddots & \vdots \\ b\bar{\beta}_{n,1} & b\bar{\beta}_{n,2} & \dots & b\bar{\beta}_{n,n}-a \\ b\bar{\beta}_{n+1,1} & b\bar{\beta}_{n+1,2} & \dots & b\bar{\beta}_{n+1,n} \end{vmatrix}$$

$$+\cdots+(-1)^{2n}b\bar{\beta}_{n,n+1}\begin{vmatrix} b\bar{\beta}_{1,1}-a & \dots & b\bar{\beta}_{1,n-1} & b\bar{\beta}_{1,n} \\ \vdots & \ddots & \vdots & \vdots \\ b\bar{\beta}_{n-1,1} & \dots & b\bar{\beta}_{n-1,n-1}-a & b\bar{\beta}_{n-1,n} \\ b\bar{\beta}_{n+1,1} & \dots & b\bar{\beta}_{n+1,n-1} & b\bar{\beta}_{n+1,n} \end{vmatrix}$$

That is,

$$|\mathbf{\Theta}_{n+1} - \psi_r\mathbf{I}_{4(n+1)}| = (-1)^{n+1}\begin{vmatrix} b\bar{\beta}_{1,1}-a & b\bar{\beta}_{1,2} & \dots & b\bar{\beta}_{1,n+1} \\ b\bar{\beta}_{2,1} & b\bar{\beta}_{2,2}-a & \dots & b\bar{\beta}_{2,n+1} \\ \vdots & \vdots & \ddots & \vdots \\ b\bar{\beta}_{n+1,1} & b\bar{\beta}_{n+1,2} & \dots & b\bar{\beta}_{n+1,n+1}-a \end{vmatrix}$$

$$= (-1)^{n+1}b^{n+1}|\bar{\boldsymbol{\beta}}_{n+1}(0) - \Lambda\mathbf{I}_{n+1}| = 0$$

Expression (S9) is therefore an eigenvalue of the matrix $\bar{\boldsymbol{\beta}}_{n+1}(0)$. The next generation matrix at the disease-free equilibrium,[4,13,14] $\Pi^* = \bar{\boldsymbol{\beta}}_{n+1}(0)d_I$, has dominant eigenvalue Λd_I which is the basic reproductive number of the epidemic system, given by Supplementary Equation (S8).

This completes the proof.

References

1. Birrell, P.J. *et al.* Bayesian modelling to unmask and predict influenza A/H1N1pdm dynamics in London. *Proc. Nat. Acad. Sci. USA* **108**, 18238-18243 (2011).

2. Miller, E. *et al.* Incidence of 2009 pandemic influenza A/H1N1 infection in England: a cross-sectional serological study. *Lancet* **375**, 1100–1108 (2010).

3. Hardelid, P. *et al.* Assessment of baseline age-specific antibody prevalence and incidence of infection to novel influenza AH1N1 2009, *Health Technol. Assess.* **14**,115-192 (2010). URL http://dx.doi.org/10.3310/hta14550-03.

4. Wearing, H. J., Rohani, P. & Keeling, M. J. Appropriate models for the management of infectious diseases. *PLOS Med.* **2**, e174 (2005).

5. Harcourt, S. E. et al. Use of a large general practice syndromic surveillance system to monitor the progress of the influenza A(H1N1) pandemic 2009 in the UK. *Epidemiol. Infect.* **140**, 100–105 (2012). URL http://dx.doi.org/10.1017/S095026881100046X

6. Health Protection Agency. (2009) Positivity rates from virological sampling. http://www.hpa.org.uk/web/HPAwebFile/HPAweb C/1259152231549

7. Mossong, J., *et al.* Social contacts and mixing patterns relevant to the spread of infectious disease. *PLoS Medicine* **5**, e74 (2008).

8. Keeling, M. J., Danon, L., Vernon, M. C. & House, T. A. Individual identity and movement networks for disease metapopulation. *Proc. Nat. Acad. Sci. USA* **107**, 8866-8870 (2010). URL http://dx.doi.org/10.1073/pnas.1000416107.

9. Eggo, R. M., Cauchemez, S. & Ferguson, N. M. Spatial dynamics of the 1918 influenza pandemic in England, Wales and the United states. *J. R. Soc. Interface.* **8**, 233-243 (2010).

10. Gog, J. R. *et al.* Spatial Transmission of 2009 Pandemic Influenza in the US. *PLoS Comput. Biol.* **10**(6), e1003635+ (2014). URL http://dx.doi.org/10.1371/journal.pcbi.1003635.

11. Ferguson, N. M. *et al.* Strategies for mitigating an influenza pandemic. *Nature* **442**, 448-452 (2006).

12. Viboud, C. *et al.* Synchrony, waves, and spatial hierarchies in the spread of influenza. *Science* **312**, 447-451 (2006). URL http://dx.doi.org/10.1126/science.1125237.

13. Vynnycky, E. & White, R. *An introduction to infectious disease modelling*. Oxford University Press, UK (2010).

14. Keeling, M. J. & Rohani, P. *Modelling Infectious Disease in Humans and Animals*. Princeton University Press, Princeton, NJ, USA (2008).

Supplementary Tables

Supplementary Table S1: Commuter matrices. For each of the four adult age groups, cells give the proportion of individuals resident in the row region, who move to (or stay in) each of the four regions.

Matrix	London	WM	North	South	London	WM	North	South
$C_{rs}(a)$	Ages: 16-24 years				Ages: 25-44 years			
London	93.50%	0.11%	0.30%	6.12%	92.10%	0.11%	0.26%	7.54%
WM	0.43%	95.60%	2.29%	1.64%	0.52%	94.80%	2.95%	1.71%
North	0.36%	0.77%	97.30%	1.57%	0.46%	0.99%	96.90%	1.67%
South	5.09%	0.22%	0.45%	94.20%	9.29%	0.30%	0.53%	89.90%
	ages: 45-64 years				ages: 65-74 years			
London	93.60%	0.10%	0.24%	6.10%	85%	0.11%	0.23%	14.60%
WM	0.37%	96%	2.32%	1.31%	0.35%	97.40%	1.41%	0.85%
North	0.35%	0.83%	97.80%	1.06%	0.34%	0.47%	98.50%	0.65%
South	6.87%	0.27%	0.46%	92.40%	4%	0.19%	0.33%	95.50%

Supplementary Table S2 Inputs for model parameters. For each parameter grouping, the table specifies the prior distributions used or, where the parameter is not to be estimated by the model, it's assumed fixed value.

Transmission model parameter	Symbol	Prior/Fixed Value
Exponential growth rates	ψ_r	~Γ(6.3, 57)
Initial log-hazards of GP consultation	υ_r	~N(−19.15, 16.44)
Mean Infectious Period	d_I	2 + Z, Z ~ Γ(518, 357)
Mean Latent Period	d_L	2
Contact matrix parameters	m_i	~ U[0, 1] ∀i
Initial proportion susceptible in age group a	π_a	1 (< 1 years), 0.980 (1-4), 0.969 (5-14), 0.845 (15-24), 0.920 (25-44), 0.865 (45-64), 0.762(65+)
**Disease and Reporting model parameters**		**Prior/Fixed Value**
Mean (s.d.) of gamma distributed incubation times		1.6(1.8)
Proportion of infections symptomatic	θ	~ β(32.5, 18.5)
Proportion of cases who consult a GP, varying by age, time and region [Note i =1,3,5,7 depending on time interval for child age classes and i = 2, 4, 6, 8 otherwise.	$p_{ra}^{(GP)}(t_n)$	$p_{ra}^{(GP)}(t_n) = \log(p_{r,i}/(1-p_{r,i}))$ $p_{i,a} \sim \begin{cases} N(-0.187, 0.166) & i = 1,2 \\ N(0.426, 0.929) & i = 3,4 \\ N(-0.319, 0.263) & i = 5,6 \\ N(-0.284, 0.264) & i = 7,8 \end{cases}$
Proportion of cases lab-confirmed	$p_r^{(CC)}$	~ β(1.03, 2.69)
Mean (s.d.) of gamma distributed waiting time from symptoms to GP consultation		2.0 (1.2)
Mean (s.d.) of gamma distributed waiting time from symptoms to lab-confirmation		6.6 (3.7)
Mean (s.d.) of gamma distributed reporting delay of GP consultations		0.5 (0.5)
Reporting delay of Lab confirmations		0
Regression parameters for the background consultation rates	$\boldsymbol{\beta}^{(B)}$	$N_{61}(\mathbf{0}, V^{(B)})$
Day of the week effects on the reporting of ILI cases, log-transformed	$\log(c_d)$	$N_6(\mathbf{0}, V^{(C)})$
GP consultation data dispersion parameters	$\eta_{r,i}$	~ Γ(0.01, 0.01)

Supplementary Table S3: Model choice. Posterior mean (and standard deviation, s.d.) deviance for some candidate parameterisations of the meta-region model, expressed as a discrepancy from the deviance of the parallel-region model. The smallest values of the posterior mean deviance represent the better fitting variants of model.

α	Density-type	Seed-type	# Strata	$\Delta D(\varphi)$ (s.d.)
0.0	By strata	nextgen	28	3,890 (34.23)
0.0	By strata	nextgen	44	4,376 (33.83)
0.0	By strata	empirical	28	4,548 (31.53)
0.0	By strata	empirical	44	4,949 (32.21)
0.5	By strata	nextgen	28	3,025 (34.21)
0.5	By strata	nextgen	44	2,241 (32.13)
0.5	By strata	empirical	28	3,191 (36.65)
0.5	By strata	empirical	44	2,269 (31.90)
1.0	By strata	nextgen	28	2,770 (30.23)
1.0	By strata	nextgen	44	2,466 (30.05)
1.0	By strata	empirical	28	2,578 (29.43)
1.0	By strata	empirical	44	2,359 (29.39)
1.0	By region	nextgen	28	449.2 (27.60)
1.0	By region	nextgen	44	437.9 (27.21)
1.0	By region	empirical	28	170.1 (28.47)
1.0	By region	empirical	44	166.4 (29.76)
1.0	By region	extended empirical	28	57.89 (27.20)
Parallel-region model				0

Supplementary Table S4 Age-specific attack rates. Table gives posterior median (and 95% CrIs) for the age-specific attack rates in each region in both waves of the 2009 pandemic. A) presents the posterior statistics from fitting the parallel-region model; and B) for the meta-region model. The last column gives attack rates averaged over four regions, weighted in accordance with the respective population sizes.

A) Parallel region model

First wave (to end-Aug)	London	West Midlands	North	South	England
Overall	13.2(11.4,14.9)	9.8(8.5,11.2)	5.6(4.4,6.9)	3.6(2.9,4.5)	6.4(5.8,7.1)
<1y	18.7(16.2,21.1)	13.6(11.8,15.4)	8.0(6.4,10.0)	5.2(4.2,6.5)	9.7(8.7,10.6)
1-4 y	29.4(25.4,33.1)	22.1(19.2,25.1)	13.2(10.4,16.5)	8.7(7.0,10.8)	15.3(13.6,17.1)
5-14y	28.4(25.0,31.4)	24.1(21.4,27.0)	13.0(10.4,16.0)	9.0(7.2,11.0)	14.9(13.5,16.4)
15-24y	11.9(10.0,13.6)	9.2(7.8,10.7)	5.6(4.4,7.0)	3.4(2.7,4.2)	6.1(5.4,6.9)
25-44y	13.0(11.1,14.8)	9.6(8.3,11.2)	5.6(4.5,7.1)	3.7(2.9,4.5)	6.7(6.0,7.5)
45-64y	7.0(5.9,8.1)	5.2(4.4,6.1)	3.1(2.4,3.9)	2.0(1.6,2.4)	3.4(3.0,3.8)
65+y	3.5(2.9,4.0)	2.9(2.4,3.4)	1.6(1.3,2.1)	1.1(0.8,1.3)	1.8(1.5,2.0)
Second wave (Sep.-Dec.)					
Overall	10.1(8.5,11.9)	10.6(9.0,12.2)	19.3(17.8,21.0)	19.6(18.3,21.0)	17.1(16.2,18.3)
<1y	13.7(11.5,16.2)	13.7(11.5,15.7)	25.6(23.6,27.6)	26.2(24.6,27.9)	22.3(21.1,23.7)
1-4 y	17.1(14.1,20.7)	18.7(15.6,21.8)	34.8(31.8,37.9)	36.7(34.3,39.2)	30.7(28.9,32.8)
5-14y	24.9(21.0,29.3)	29.2(24.9,33.5)	50.4(46.8,53.9)	53.5(50.6,56.1)	45.6(43.5,47.7)
15-24y	9.5(7.9,11.2)	10.0(8.4,11.6)	19.4(17.6,21.3)	18.8(17.3,20.6)	16.7(15.5,18.2)
25-44y	9.5(7.9,11.2)	9.0(8.4,11.5)	18.6(16.9,20.4)	18.8(17.4,20.4)	16.0(15.0,17.4)
45-64y	5.4(4.5,6.4)	5.5(4.7,6.5)	10.7(9.7,11.8)	10.5(9.7,11.6)	9.4(8.7,10.3)
65+y	3.4(2.8,4.0)	3.6(3.1,4.2)	6.9(6.3,7.6)	6.9(6.3,7.5)	6.1(5.7,6.7)

B) Meta-region model

First wave (to end-Aug)	London	West Midlands	North	South	England
Overall	9.9(8.9,11.0)	12.4(11.5,13.3)	4.7(4.2,5.2)	6.0(5.4,6.6)	6.8(6.1,7.4)
<1y	14.0(12.6,15.4)	16.9(15.7,18.1)	6.5(5.8,7.2)	8.4(7.6,9.3)	9.7(8.8,10.6)
1-4 y	20.6(17.9,23.4)	25.4(22.6,28.0)	9.9(8.4,11.4)	12.7(11.0,14.5)	14.4(12.5,16.3)
5-14y	20.8(19.0,22.7)	29.6(27.6,31.6)	10.4(9.5,11.5)	13.8(12.6,15.1)	15.3(14.0,16.6)
15-24y	9.3(8.1,10.5)	12.2(11.2,13.3)	4.9(4.3,5.6)	6.0(5.4,6.8)	6.7(6.0,7.5)
25-44y	10.0(8.9,11.2)	12.5(11.6,13.6)	4.9(4.3,5.4)	6.4(5.7,7.1)	7.1(6.4,7.9)
45-64y	5.4(4.8,6.1)	7.0(6.4,7.6)	2.7(2.4,3.1)	3.5(3.1,3.9)	3.8(3.4,4.3)
65+y	2.7(2.3,3.0)	4.0(3.6,4.3)	1.4(1.3,1.6)	1.9(1.7,2.2)	2.0(1.8,2.3)
Second wave (Sep.-Dec.)					
Overall	16.2(14.9,17.6)	8.9(7.5,10.4)	20.6(19.6,21.7)	18.0(17.0,19.0)	17.8(16.7,18.9)
<1y	21.7(19.9,23.5)	11.1(9.4,13.0)	26.7(25.4,28.0)	23.5(22.1,24.8)	23.0(21.6,24.4)
1-4 y	28.0(25.2,30.9)	15.1(12.5,18.0)	37.0(35.0,39.2)	32.6(30.4,34.8)	31.5(29.3,33.8)
5-14y	36.7(34.0,39.5)	22.0(18.8,25.5)	51.8(50.2,53.5)	46.7(44.7,48.6)	44.5(42.5,46.6)
15-24y	15.8(14.5,17.3)	9.0(7.6,10.6)	21.2(19.7,22.6)	17.9(16.7,19.3)	17.9(16.6,19.3)
25-44y	15.6(14.3,17.0)	8.7(7.3,10.2)	20.2(18.9,21.4)	17.7(16.5,18.9)	17.3(16.0,18.5)
45-64y	9.1(8.3,10.0)	5.0(4.2,5.9)	11.7(10.8,12.6)	10.2(9.4,11.0)	10.1(9.3,10.9)
65+y	5.6(5.1,6.2)	3.3(2.7,3.9)	7.6(7.0,8.1)	6.7(6.2,7.2)	6.5(6.0,7.1)

Supplementary Figures

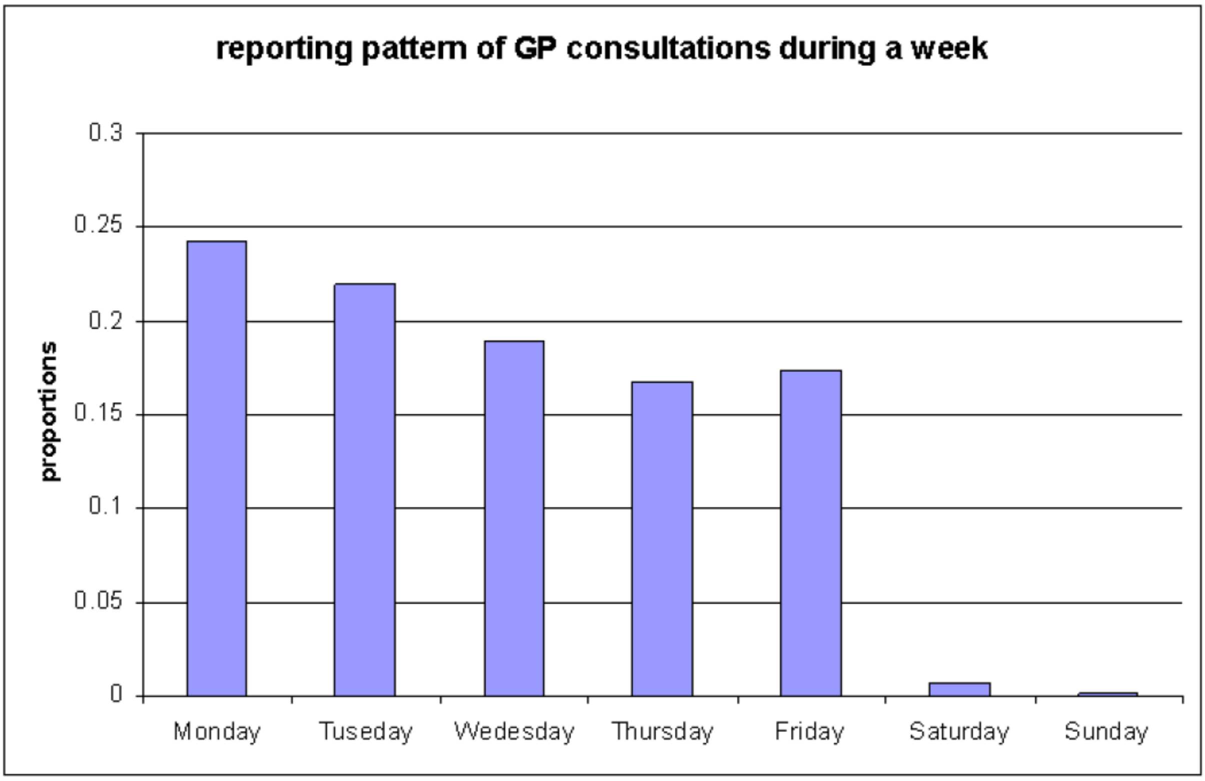

Supplementary Figure S1: *Daily reporting patterns of GP consultations within London*. The bars give the proportion of the total number of consultations that were reported on each of the days of the week. As can be seen, consultations are typically not reported on a Saturday or a Sunday.

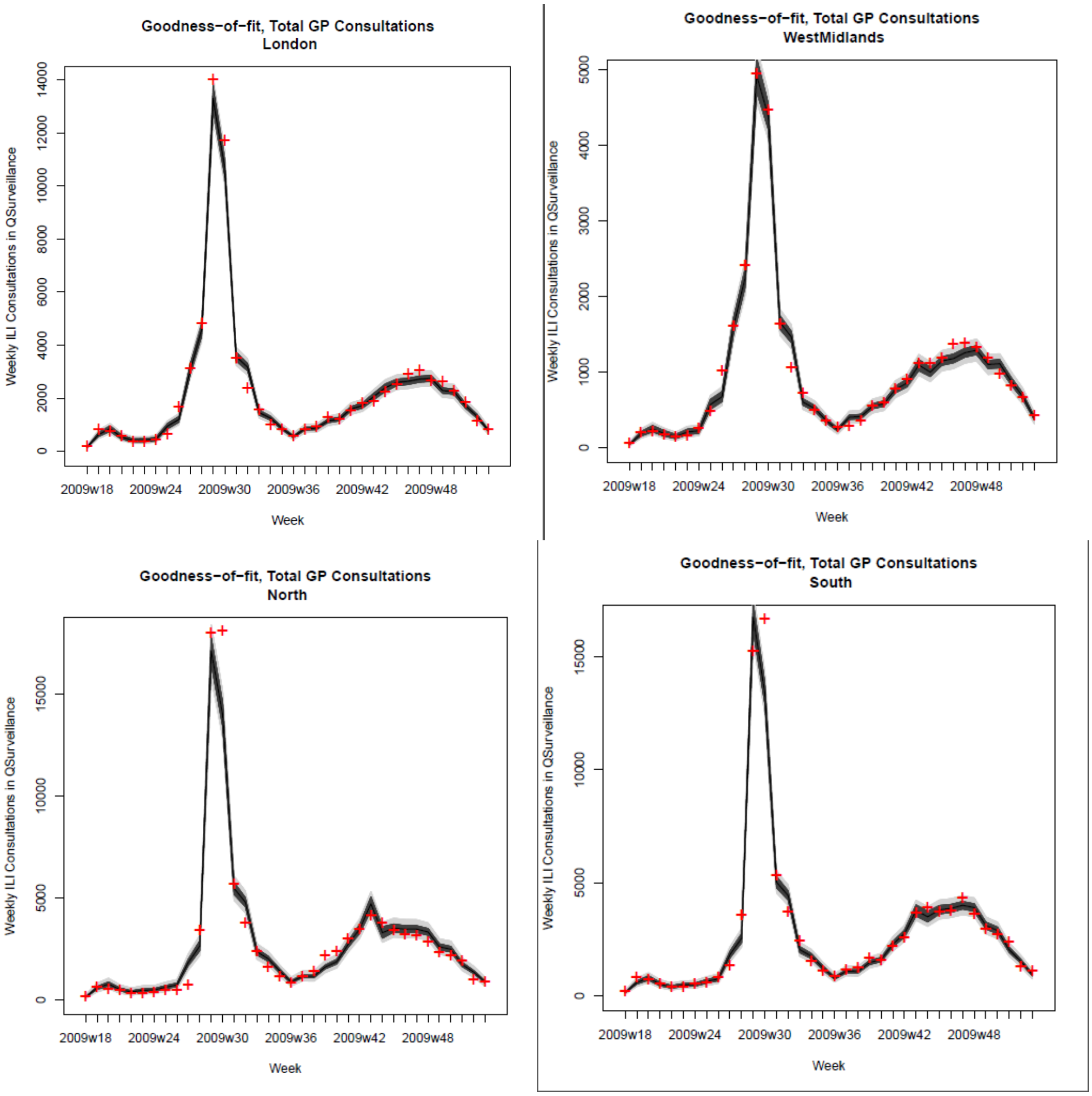

Supplementary Figure S2 *Goodness-of-fit of the parallel-region model to the GP consultation data, aggregated by age*. Red +'s indicate the observed number of consultations. The darker shaded area represent the 95% CrI for the **expected** number of consultations, the wider, lighter grey interval gives a posterior predictive 95% CrI for the observed data – i.e. 95% of the data points should lie within this wider interval.

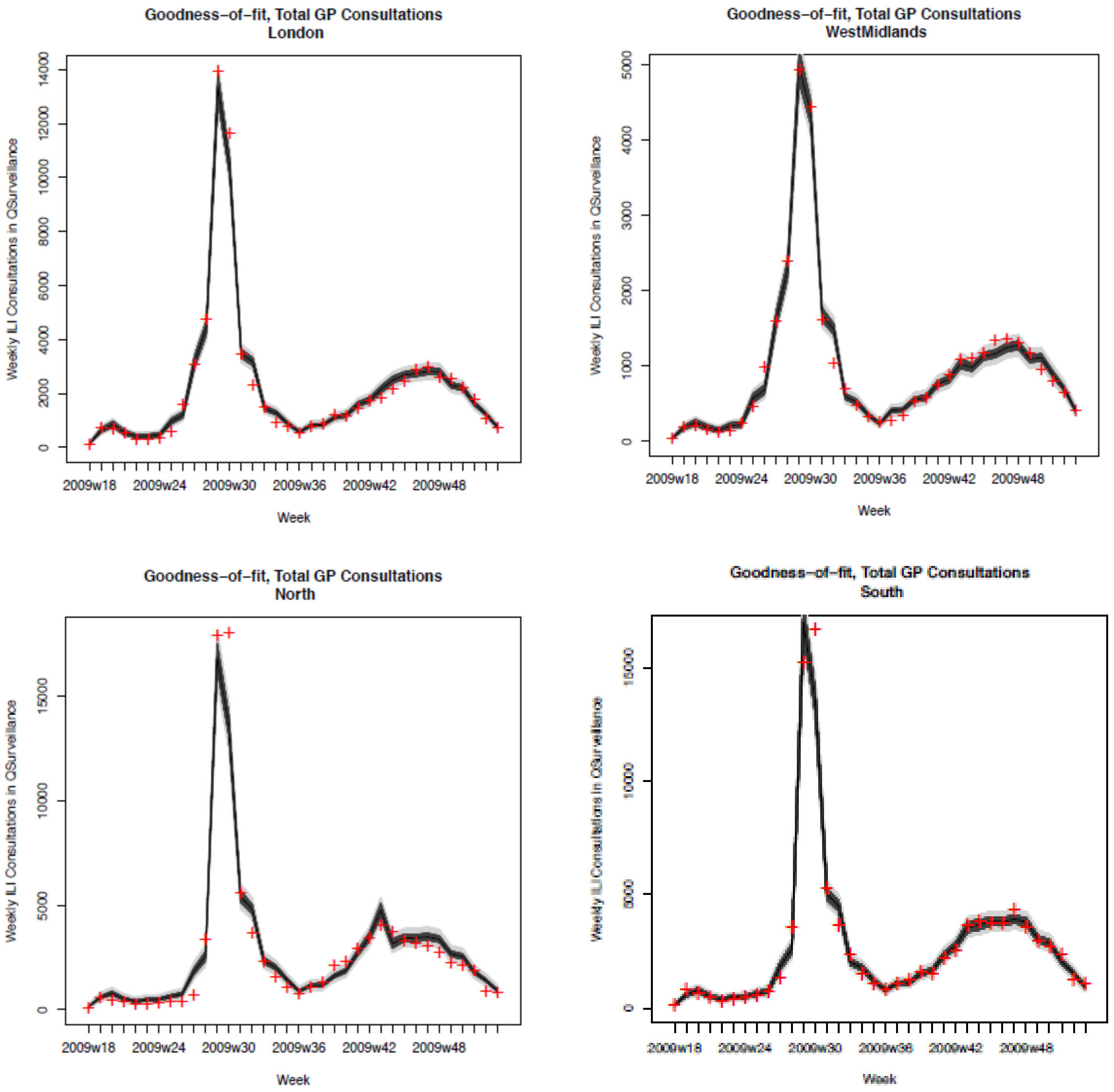

Supplementary Figure S3 *Goodness-of-fit of the meta-region model to the GP consultation data, aggregated by age*. See Supplementary Figure S2 for explanation.

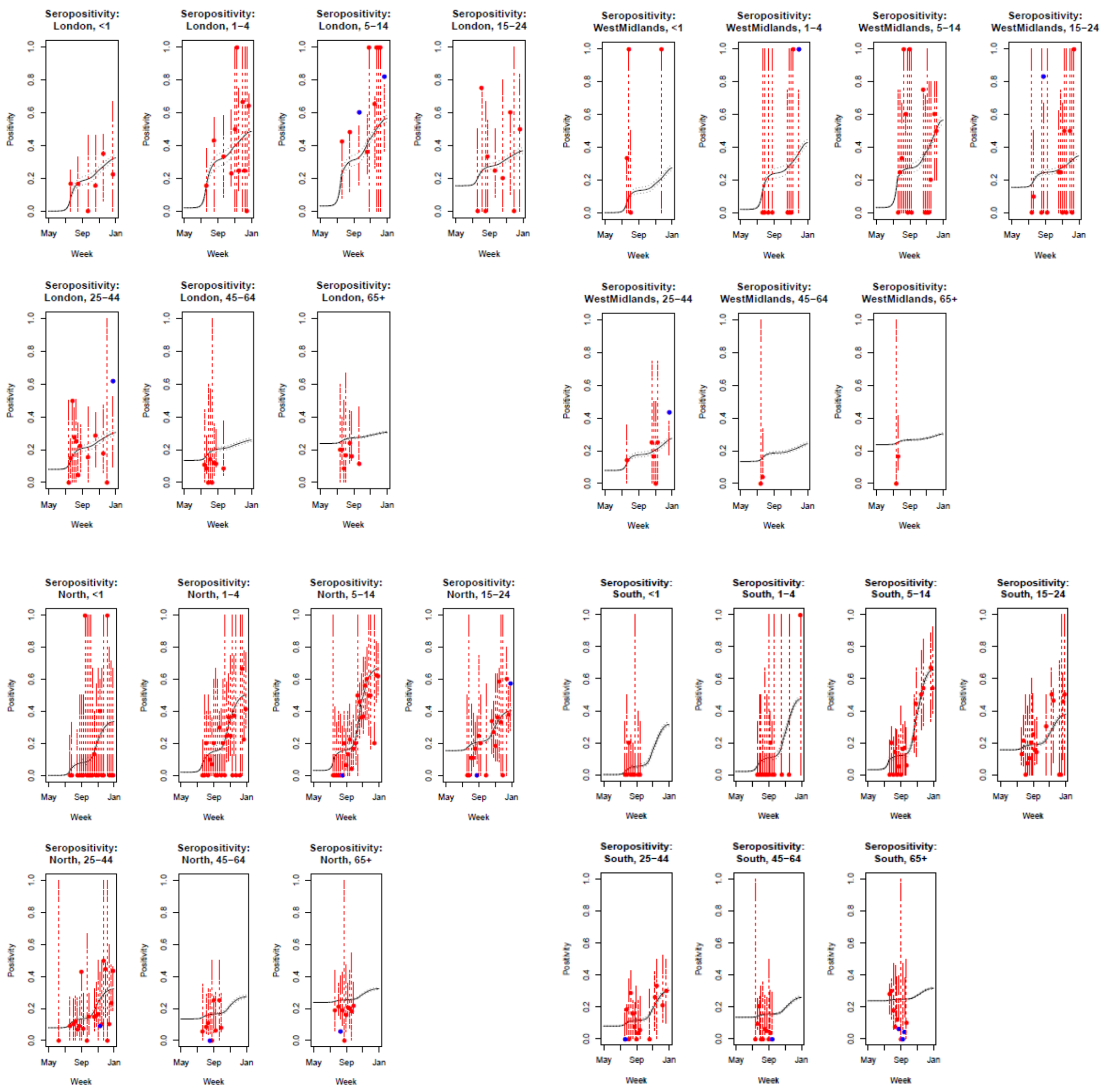

Supplementary Figure S4(a) *Goodness-of-fit of the parallel-region model to the seropositivity data, stratified by age and region*. Data points are marked by the dots, with blue dots being those that are omitted by the model predicted 95% credible intervals (the vertical dashed lines).

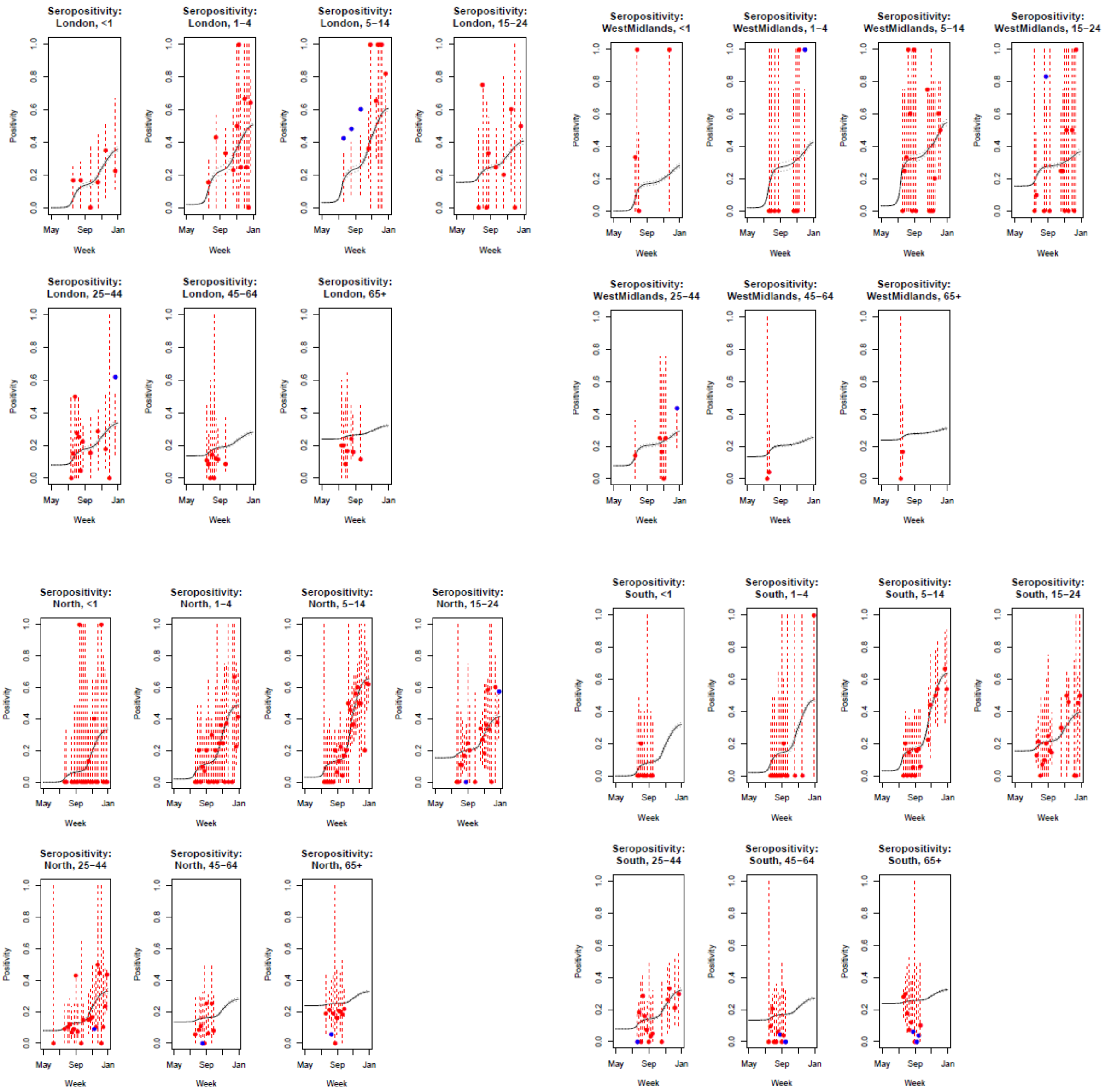

Supplementary Figure S4(b) *Goodness-of-fit of the meta-region model to the seropositivity data, stratified by age and region*. See Supplementary Figure S4(a) for explanation.

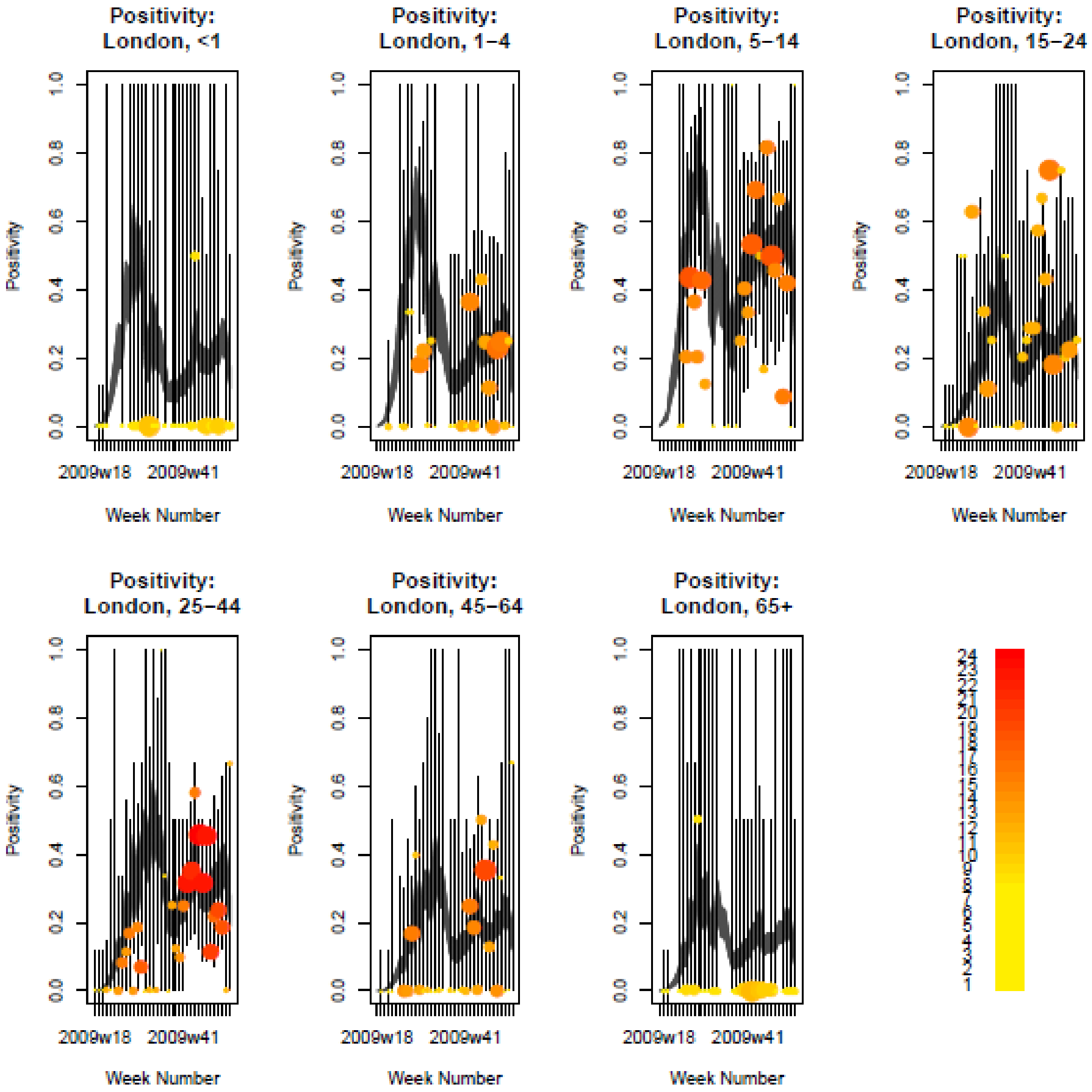

Supplementary Figure S5(a) *Goodness-of-fit of the parallel-region model to the weekly-aggregated viropositivity data, stratified by age in London*. Data points are given by the dots of variable with and colour. The width indicates the size of the denominator relative to other points in the same plot. The colour of the points indicates the overall size of the denominator, with dark red points being those of largest sample size. The light grey vertical lines give a containing 95% CrI for the data points under the model.

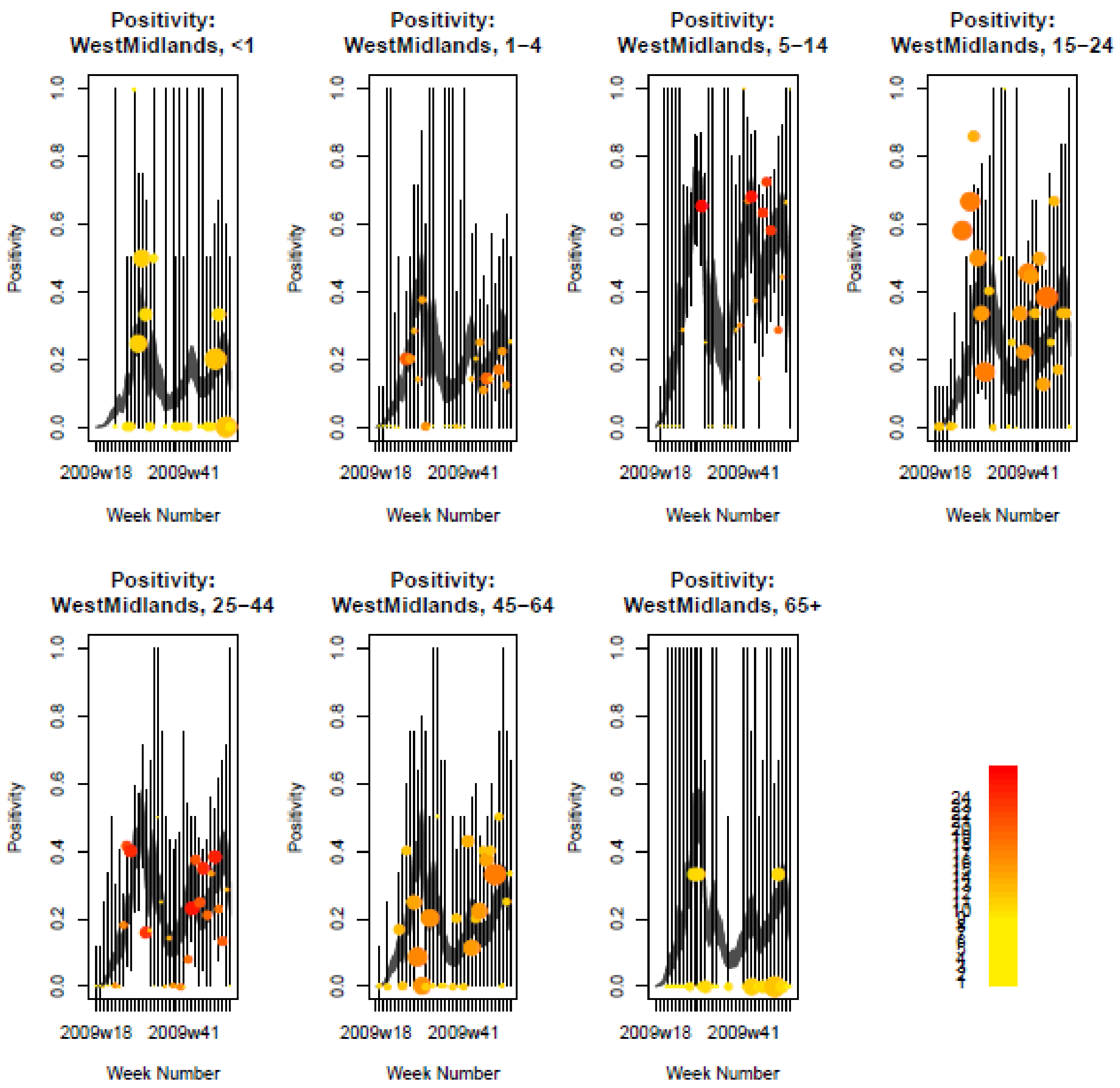

Supplementary Figure S5(b) *Goodness-of-fit of the parallel-region model to the weekly-aggregated viropositivity data, stratified by age in the West Midlands*. See Supplementary Figure S5(a) for greater detail.

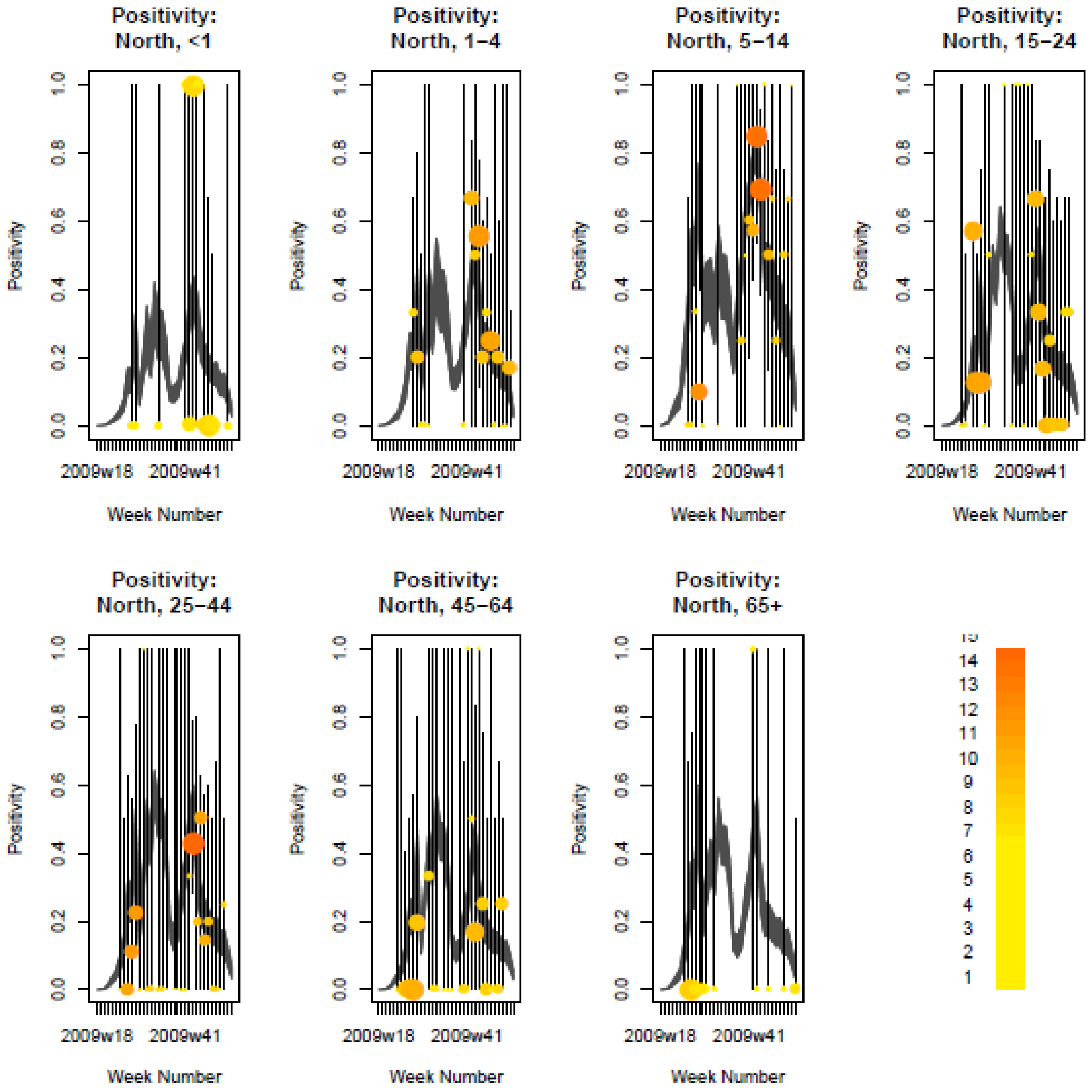

Supplementary Figure S5(c) *Goodness-of-fit of the parallel-region model to the weekly-aggregated viropositivity data, stratified by age in the North*. See Supplementary Figure S5(a) for greater detail.

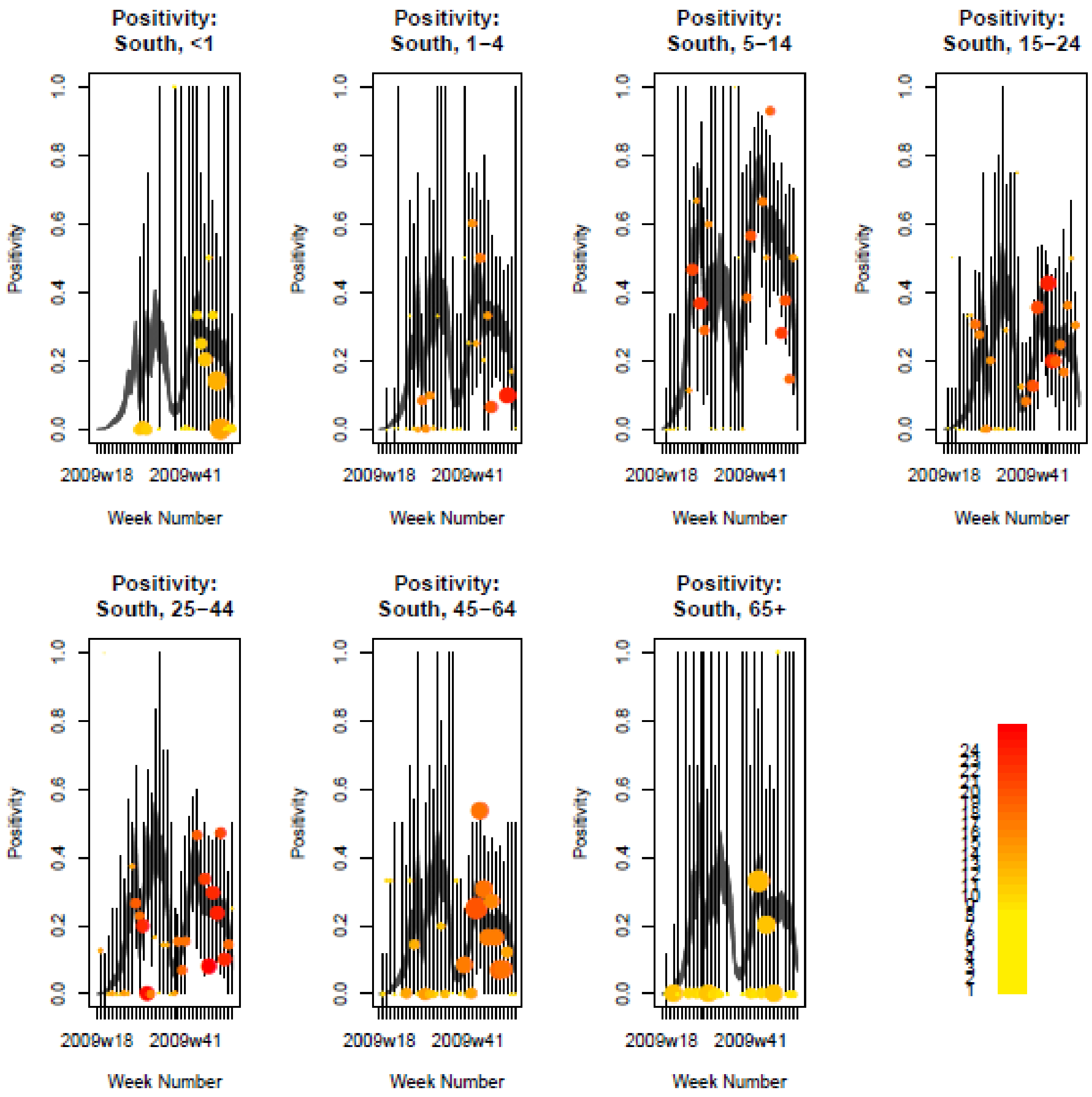

Supplementary Figure S5(d) *Goodness-of-fit of the parallel-region model to the weekly-aggregated viropositivity data, stratified by age in the South*. See Supplementary Figure S5(a) for greater detail.

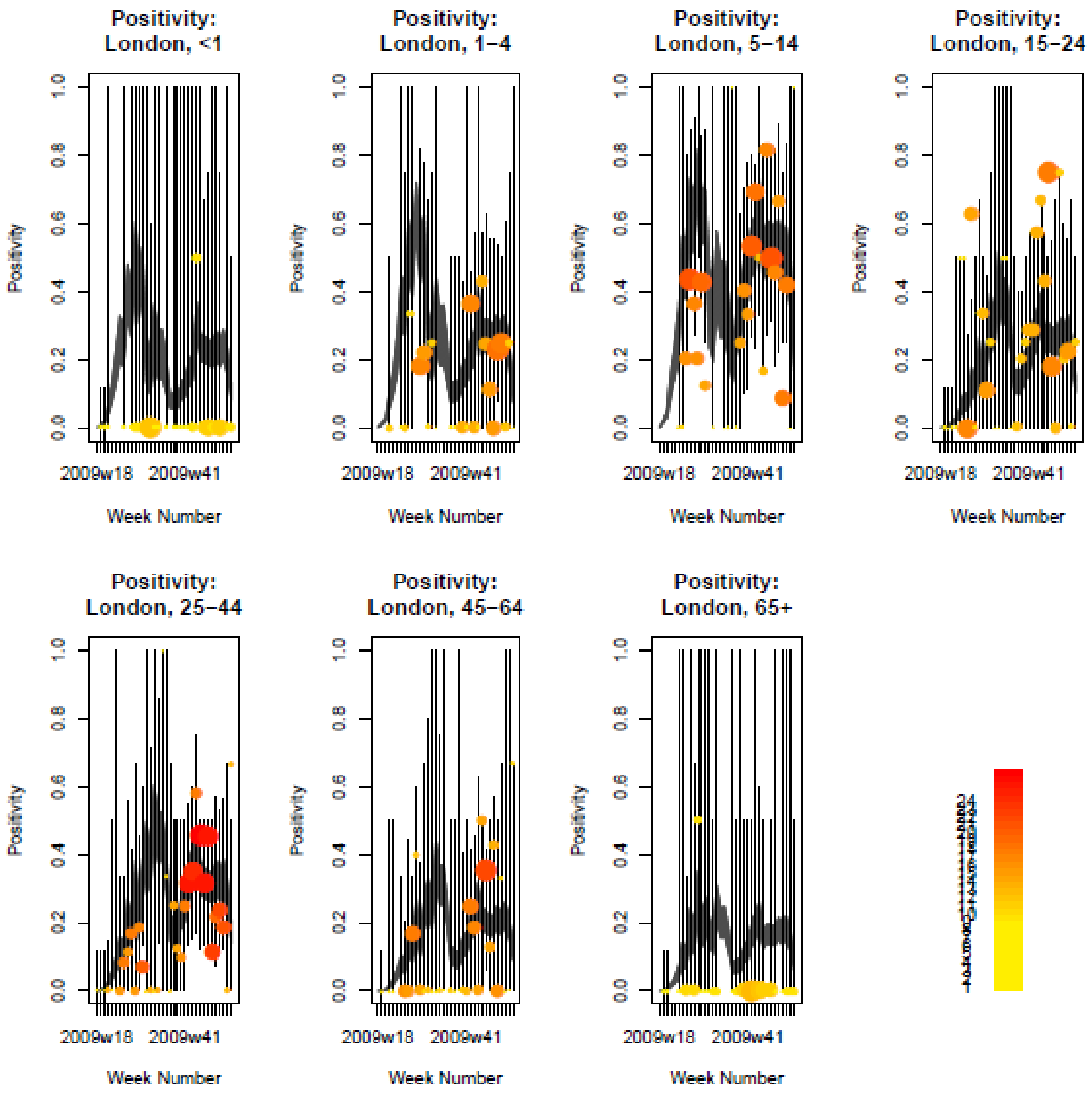

Supplementary Figure S5(e) *Goodness-of-fit of the meta-region model to the viropositivity data, stratified by age in London.* See Supplementary Figure S5(a) for a more in-depth explanation.

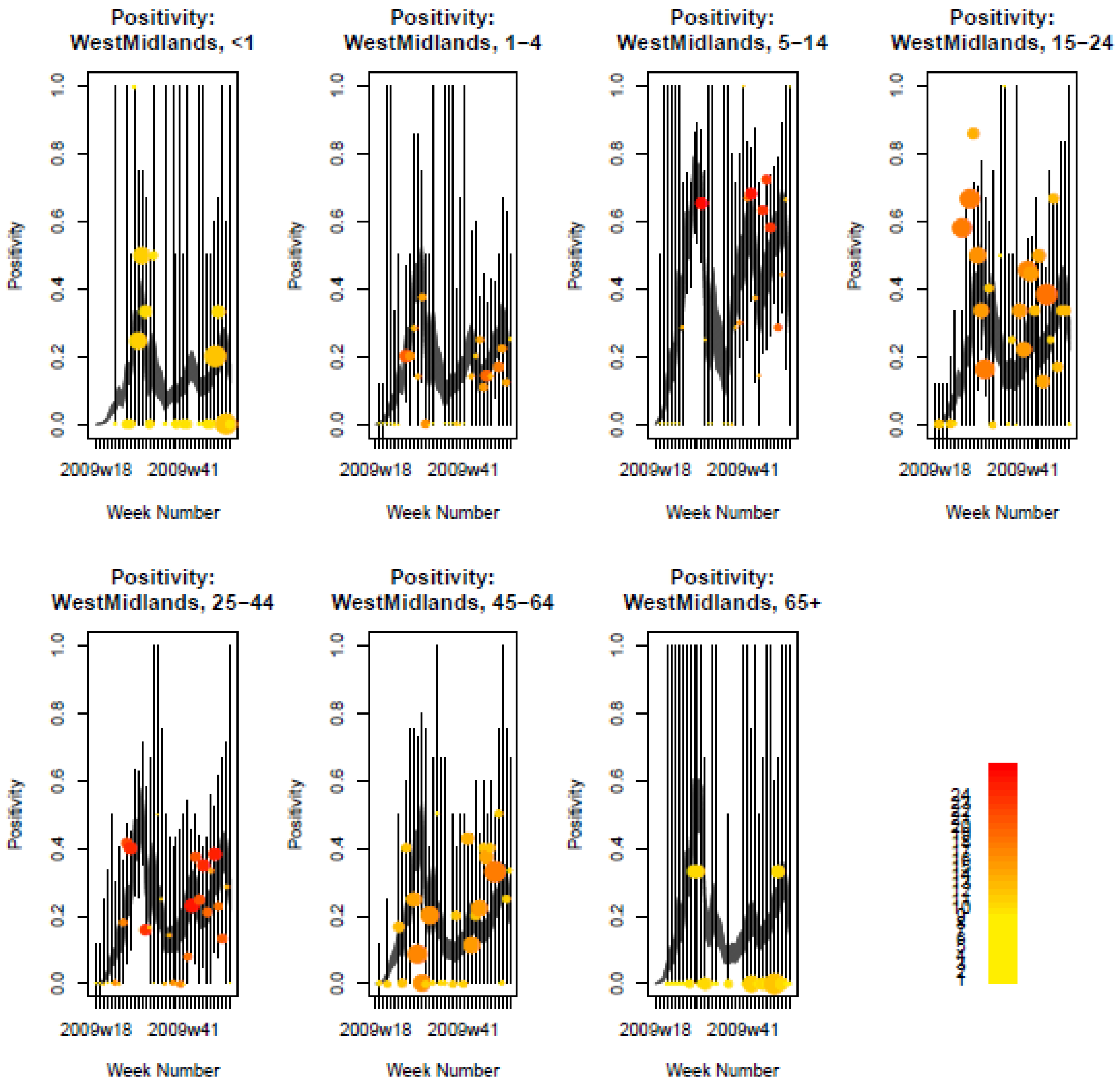

Supplementary Figure S5(f) *Goodness-of-fit of the meta-region model to the viropositivity data, stratified by age in the West Midlands.* See Supplementary Figure S5(a) for a more in-depth explanation.

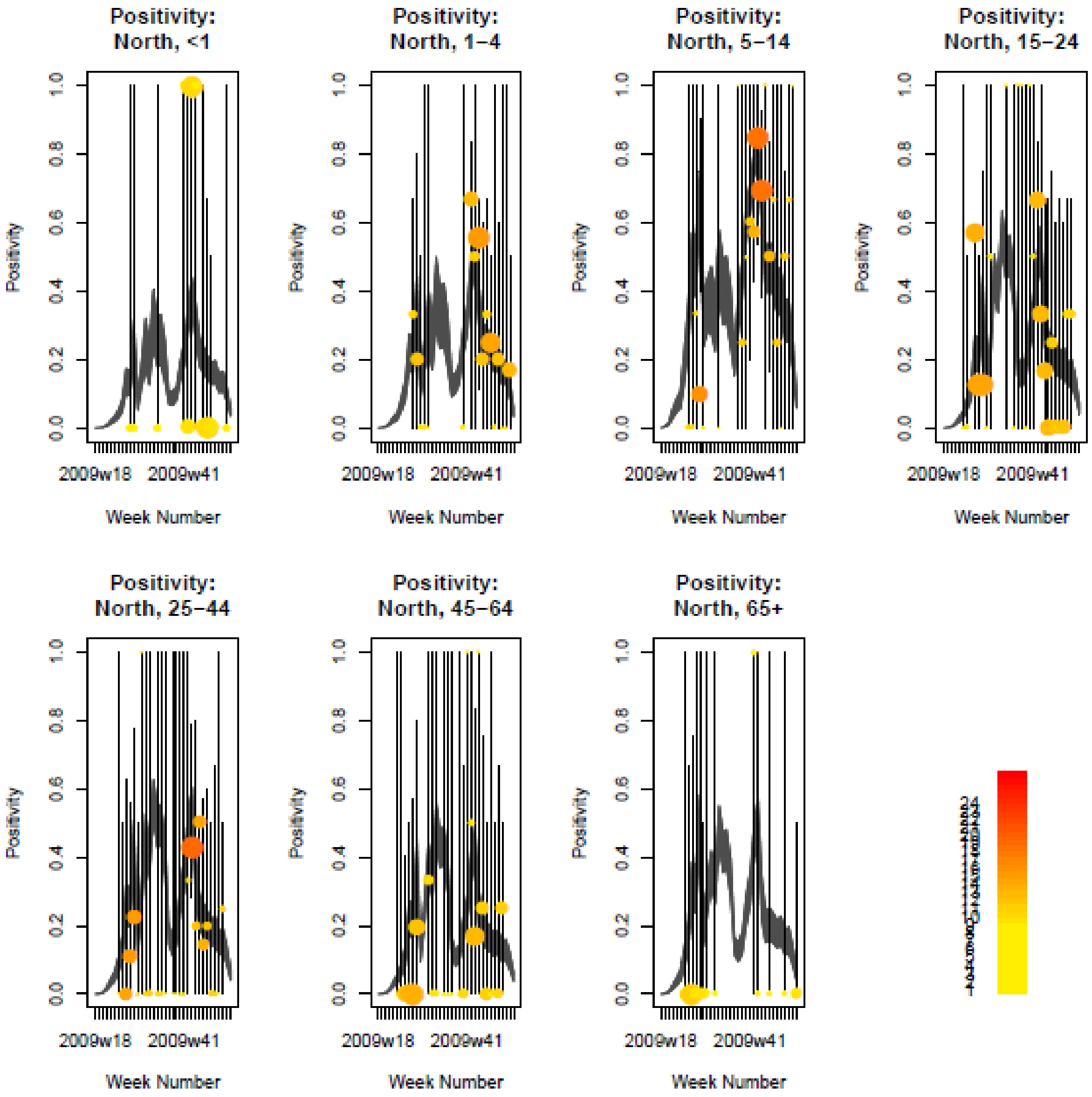

Supplementary Figure S5(g) *Goodness-of-fit of the meta-region model to the viropositivity data, stratified by age in the North.* See Supplementary Figure S5(a) for a more in-depth explanation.

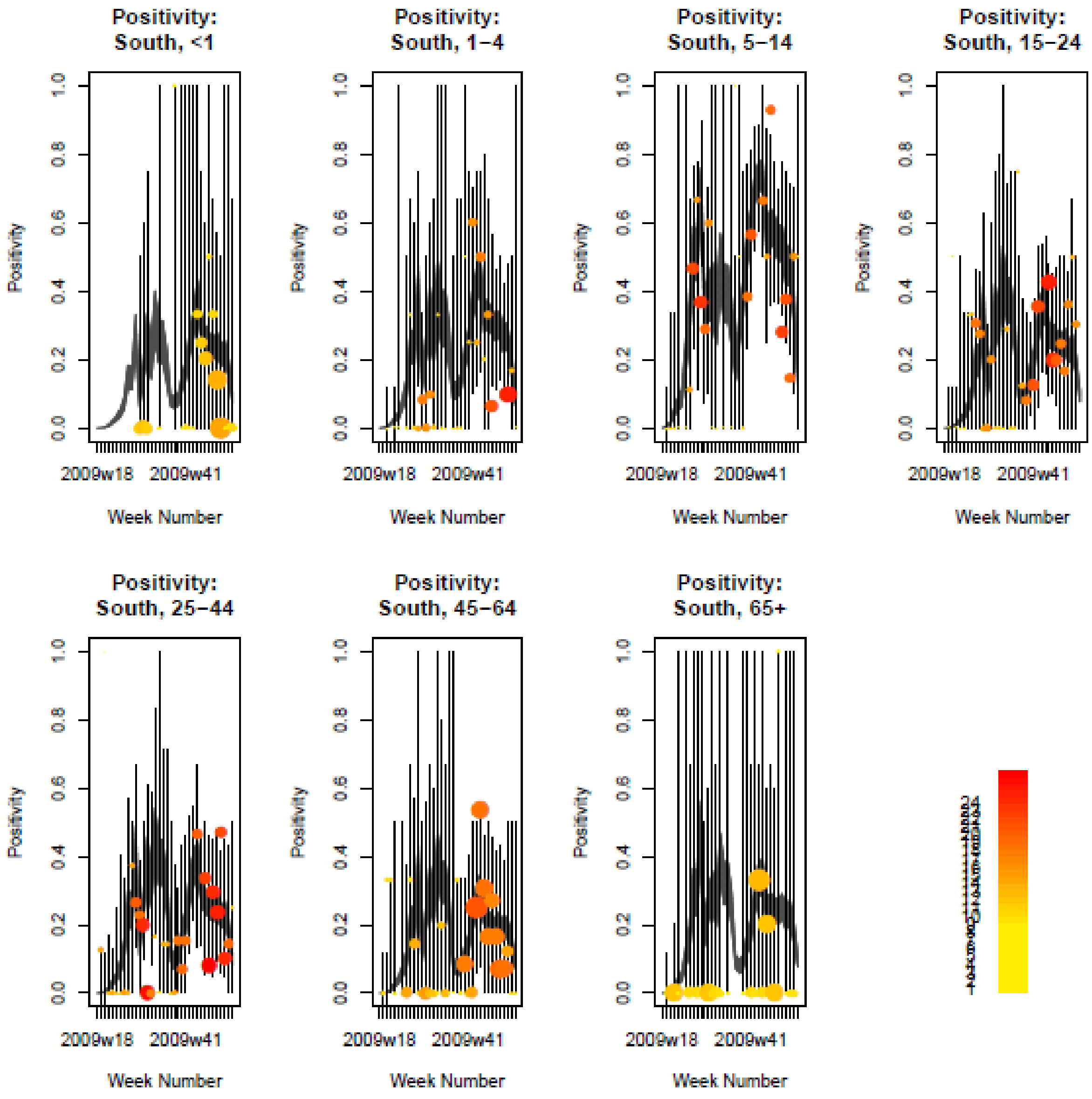

Supplementary Figure S5(h) *Goodness-of-fit of the meta-region model to the viropositivity data, stratified by age in the South.* See Supplementary Figure S5(a) for a more in-depth explanation.
